# TGF-**β**1 induces PD-1 expression in macrophages through SMAD3/STAT3 cooperative signaling in chronic inflammation

**DOI:** 10.1172/jci.insight.165544

**Published:** 2024-03-05

**Authors:** Zhigang Lei, Rui Tang, Yu Wu, Chenxu Mao, Weijie Xue, Junyao Shen, Jiaojiao Yu, Xiaohong Wang, Xin Qi, Chuan Wei, Lei Xu, Jifeng Zhu, Yalin Li, Xiujun Zhang, Chunyan Ye, Xiaojun Chen, Xiaojun Yang, Sha Zhou, Chuan Su

**Affiliations:** 1 State Key Laboratory of Reproductive Medicine and Offspring Health, National Vaccine Innovation Platform of Nanjing Medical University, Jiangsu Key Laboratory of Pathogen Biology, Department of Pathogen Biology and Immunology, School of Basic Medical Sciences, Nanjing Medical University, Nanjing, China.; 2 Department of Tropical Infectious Diseases, Naval Medical University, Shanghai, China.; 3 Department of Liver Diseases, Institute of Hepatology, the Third People’s Hospital of Changzhou, Changzhou Medical Center, Nanjing Medical University, Changzhou, China.; 4 Department of General Surgery, the Second Affiliated Hospital of Nanjing Medical University, Nanjing, China.; 5 Department of General Surgery, the Friendship Hospital of Ili Kazak Autonomous Prefecture, Yining, Xinjiang Uygur Autonomous Region, China.

**Keywords:** Immunology, Inflammation, Macrophages

## Abstract

Programmed cell death protein 1 (PD-1), a coinhibitory T cell checkpoint, is also expressed on macrophages in pathogen- or tumor-driven chronic inflammation. Increasing evidence underscores the importance of PD-1 on macrophages for dampening immune responses. However, the mechanism governing PD-1 expression in macrophages in chronic inflammation remains largely unknown. TGF-β1 is abundant within chronic inflammatory microenvironments. Here, based on public databases, significantly positive correlations between *PDCD1* and *TGFB1* gene expression were observed in most human tumors. Of note, among immune infiltrates, macrophages as the predominant infiltrate expressed higher *PDCD1* and *TGFBR1/TGFBR2* genes. MC38 colon cancer and *Schistosoma* *japonicum* infection were used as experimental models for chronic inflammation. PD-1^hi^ macrophages from chronic inflammatory tissues displayed an immunoregulatory pattern and expressed a higher level of TGF-β receptors. Either TGF-β1–neutralizing antibody administration or macrophage-specific *Tgfbr1* knockdown largely reduced PD-1 expression on macrophages in animal models. We further demonstrated that TGF-β1 directly induced PD-1 expression on macrophages. Mechanistically, TGF-β1–induced PD-1 expression on macrophages was dependent on SMAD3 and STAT3, which formed a complex at the *Pdcd1* promoter. Collectively, our study shows that macrophages adapt to chronic inflammation through TGF-β1–triggered cooperative SMAD3/STAT3 signaling that induces PD-1 expression and modulates macrophage function.

## Abstract 2

Macrophages adapt to chronic inflammation through TGF-&beta;1-triggered cooperative signaling of SMAD3-STAT3 that induces and maintains a high level of PD-1 expression.

## Graphical abstract

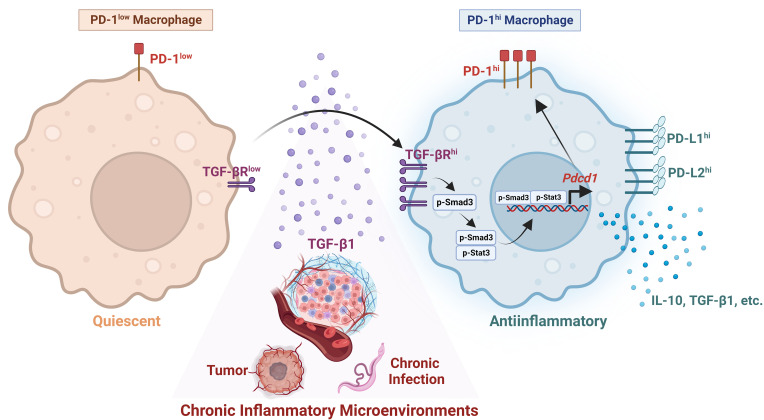

## Introduction

The inhibitory receptor programmed cell death 1 (PD-1; also called CD279 in humans) is a major mediator of peripheral immune tolerance. PD-1 engagement by its ligands, PD-L1 or PD-L2, transmits inhibitory signals into immune cells via an immunoreceptor tyrosine–based inhibitory motif and an immunoreceptor tyrosine–based switch motif located in its cytoplasmic tail. The great success of immunotherapy with PD-1 blockade in various types of cancers supports the essential role of PD-1 in immune suppression. The initial emphasis on PD-1–mediated immunosuppression has been predominantly placed on T effector cells. Activation of the PD-1 pathway inhibits T cell proliferation, cytokine production, cytolytic function, and survival in chronic infections and cancers (1–3).

Surprisingly, compared with T cell–specific deletion of PD-1, myeloid specific PD-1 deletion led to more effective inhibition of tumor growth in various tumor models, as effective as global deletion of PD-1 (4–8). These remarkable results highlight the importance of PD-1 on myeloid cells in mediating immunosuppression. Notably, macrophages derived from myeloid progenitors highly express PD-1, arising in settings of immunosuppressive tissue microenvironment, such as pathogen- and tumor-driven chronic inflammation. The ligation of PD-1 on macrophages by PD-L1/PD-L2 promotes dysfunctional or tolerogenic macrophages, which in turn reduces T cell activation and limits immune-mediated clearance of pathogens or degenerated cells (9–16).

Studies have shown that pathogen- or tumor-driven chronic inflammation induces and sustains a high level of PD-1 expression on immune cells. Previous studies have focused on the molecular mechanisms driving PD-1 expression in T cells and have shown that T cell receptor (TCR) stimulation is necessary to initiate a signaling cascade, resulting in activation and translocation of nuclear factor of activated T cell 1 (NFATc1). The binding of NFATc1 to a conserved region (CR-C) within the *Pdcd1* promoter is required for initial PD-1 expression in CD4^+^ and CD8^+^ T cells upon activation (17–20). In addition, TCR- and NFATc1-dependent PD-1 expression in T cells is further augmented by multiple cytokines, such as TGF-β1, IL-6, or IL-12 (19, 20).

Macrophages are innate immune cells with differences in antigen recognition, activation (21), and effects and may use different pathways to regulate PD-1 expression. To date, not much is known about the regulation of PD-1 expression on macrophages. In an acute inflammation microenvironment, following an encounter with pathogen-derived TLR ligands or inflammatory cytokines (IFN-α, TNF-α, etc.), macrophages initiate PD-1 expression by NF-κB or STATs (9, 22). However, macrophages with an antiinflammatory phenotype, which are predominantly found in chronic infections or tumors, tend to permanently express much higher levels of PD-1 (14, 15, 23), which indicates that there may exist different mechanisms governing PD-1 expression in macrophages under antiinflammatory conditions.

Here, we provide both clinical relevance and experimental evidence to support that TGF-β1, which plays a vital role in suppressing immune functions, directly and sufficiently induced *Pdcd1* gene transcription through a SMAD3/STAT3 complex at the *Pdcd1* promoter in macrophages, resulting in maintaining high expression of PD-1 on macrophages in chronic inflammation. Our results not only reveal a critical role and mechanism for TGF-β1 in the induction of PD-1 expression on macrophages but also suggest a potential therapeutic strategy for regulating PD-1 expression on macrophages in chronic inflammation.

## Results

### Transcriptomic profile of PD-1^hi^ macrophages in chronic inflammatory tissues.

**Figure 1:**
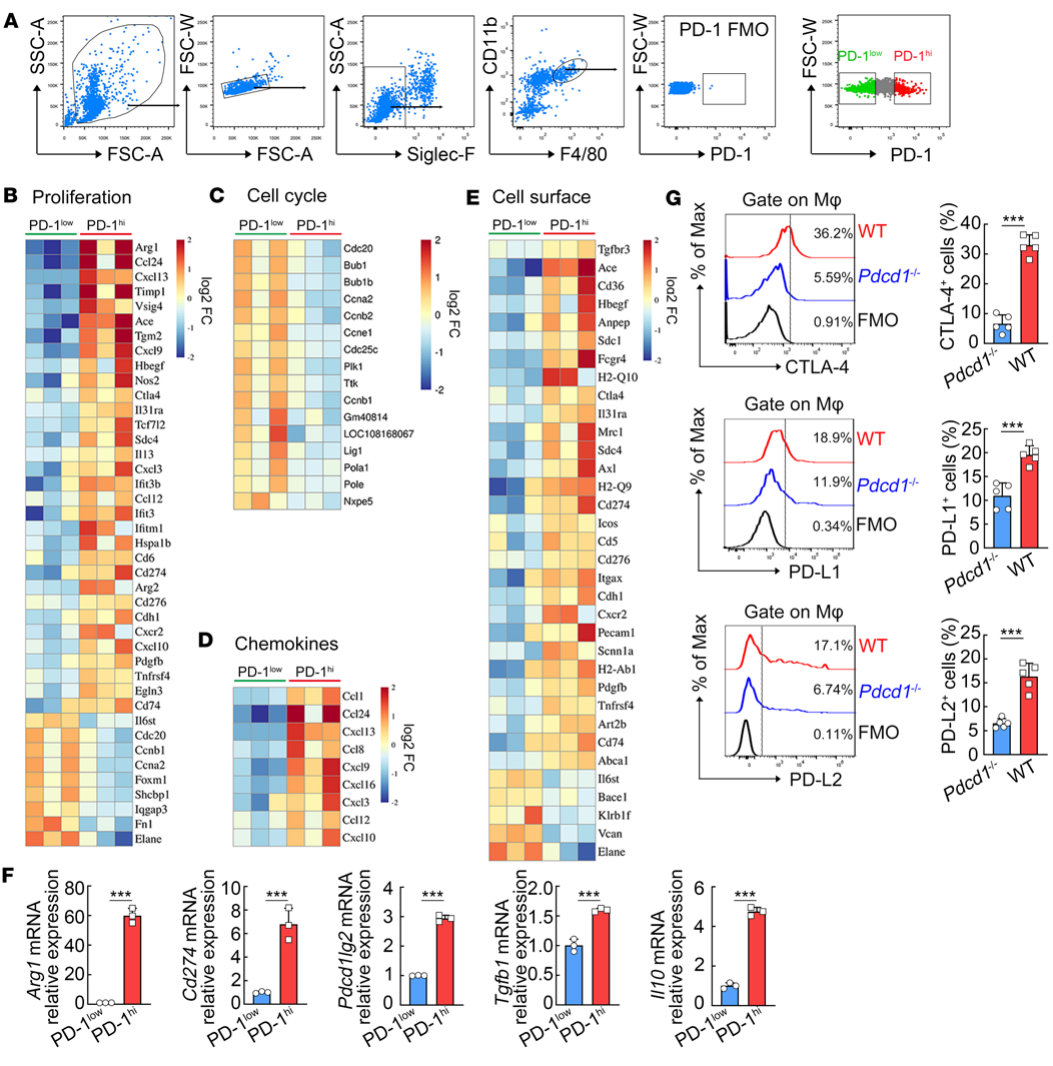
Transcriptomic profile of PD-1^hi^ macrophages in chronic inflammatory tissues. C57BL/6 mice were infected with *S*. *japonicum*. At 8 weeks after *S*. *japonicum* infection, macrophages were isolated from livers for further analysis. (**A**) The gating strategy for sorting PD-1^lo^ and PD-1^hi^ macrophages (SiglecF^–^CD11b^+^F4/80^+^) is shown, excluding the macrophages expressing medium levels of PD-1 (PD-1^med^; gray dots on the plot) according to the fluorescence minus one (FMO) control for PD-1 expression. (**B**–**E**) Heatmaps showing the corresponding log_2_ fold-changes in DEG RNA-Seq expression values in the comparison of PD-1^hi^ versus PD-1^lo^ macrophages, based on cell proliferation (**B**), cell cycle (**C**), chemokines (**D**), and cell surface protein (**E**) cluster analysis. (**F**) The relative mRNA levels of *Arg1*, *Cd274*, *Pdcd1lg2*, *Tgfb1*, and *Il10* in PD-1^lo^ and PD-1^hi^ macrophages were determined using RT-PCR. (**G**) *Pdcd1*^+/+^ (WT) and *Pdcd1*^–/–^ mice were infected with *S*. *japonicum*. At 8 weeks postinfection, liver mononuclear cells were isolated. Cytotoxic T lymphocyte-associated antigen-4 (CTLA-4), PD-L1, and PD-L2 on macrophages were analyzed by FCM. Representative histograms are shown and bar graphs show the quantification of CTLA-4^+^, PD-L1^+^, or PD-L2^+^ macrophages. An unpaired 2-tailed *t* test (**F** and **G**) was used for statistical analysis. The data are expressed as the mean ± SD of 3 or 5 mice per group. ***P < 0.001.

To investigate the global gene expression profile of expressing PD-1^hi^ macrophages in chronic inflammatory tissues, we performed RNA sequencing (RNA-Seq) to compare gene expression patterns between PD-1^hi^ and PD-1^lo^ macrophages (Figure 1A), both of which were purified from livers of *Schistosoma* *japonicum*–infected mice, a well-defined model for chronic tissue inflammation. Between PD-1^hi^ and PD-1^lo^ macrophages, a total of 941 differentially expressed genes (DEGs) were identified, and 284 DEGs showed equal or greater than 2-fold change (*P* < 0.05, Supplemental Figure 1A; supplemental material available online with this article; https://doi.org/10.1172/jci.insight.165544DS1). Of the 284 DEGs, 164 were upregulated and 120 downregulated in PD-1^hi^ macrophages (Supplemental Figure 1B). Furthermore, the Gene Ontology (GO) analysis revealed that the DEGs of PD-1^hi^ macrophages were enriched in response to stress, immune system process, immune response, response to cytokine, cell surface, response to stimulus, innate immune response, and so on (Supplemental Figure 1C). Subsequently, the DEGs between PD-1^hi^ and PD-1^lo^ macrophages were subjected to Kyoto Encyclopedia of Genes and Genomes (KEGG) pathway enrichment analysis (Supplemental Figure 1D). Following visualizing the overall pattern of gene expression at the single-gene level, PD-1 was found to be associated with the expression of transcripts in macrophages involved in proliferation (Figure 1B), cell cycle (Figure 1C), chemokines (Figure 1D), and cell surface molecules (Figure 1E). Specifically, compared with PD-1^lo^ macrophages, the expression of genes encoding positive regulators of cell proliferation and cycle (e.g., *Cdc20*, *Ccna2*, *Foxm1*, *Plk1*, *Ttk*) was lower in PD-1^hi^ macrophages; the expression of genes encoding negative regulators of immune responses (e.g., *Arg1*, *Ctla4*, *Cd274*, *Cd276*) was higher in PD-1^hi^ macrophages (Figure 1, B–E).

We next performed RT-PCR to further verify the immunomodulatory phenotype of PD-1^hi^ macrophages. The mRNA expression levels of negative immune regulators (*Arg1*, *Cd274*, *Pdcd1lg2*, *Tgfb1*, and *Il10*) were significantly higher in PD-1^hi^ macrophages compared with PD-1^lo^ macrophages (Figure 1F). Interestingly, flow cytometry analysis further showed that macrophages in *S*. *japonicum*–infected PD-1–deficient mice expressed significantly lower levels of other immune-inhibitory molecules that can convey immunosuppressive functions, such as CTLA-4, PD-L1, and PD-L2, compared with macrophages in their WT controls (Figure 1G), suggesting an association of PD-1 with the immunoregulatory phenotype of macrophages. The tumor microenvironment is also characterized by chronic inflammation (24). Based on The Cancer Genome Atlas (TCGA) database (Gene Expression Profiling Interactive Analysis [GEPIA] platform), M2 macrophages with immunoregulatory properties were the most abundant immune cells infiltrating in common human tumors (colon, lung, and stomach adenocarcinomas; Supplemental Figure 2A) and possessed a preferential expression of *Pdcd1* gene (Supplemental Figure 2B). Further database analysis (TIMER2.0) showed that *PDCD1* gene expression was positively correlated with the levels of immunosuppressive genes (*CD274*, *PDCD1LG2*, *TGFB1*, and *IL10*) in most human tumor types (Supplemental Figure 2C).

These data suggest that PD-1^hi^ macrophages in chronic inflammatory tissues possess a unique transcriptomic profile and display an immunoregulatory pattern.

### TGF-β1 is involved in inducing PD-1 expression on macrophages in chronic inflammatory tissues.

**Figure 2:**
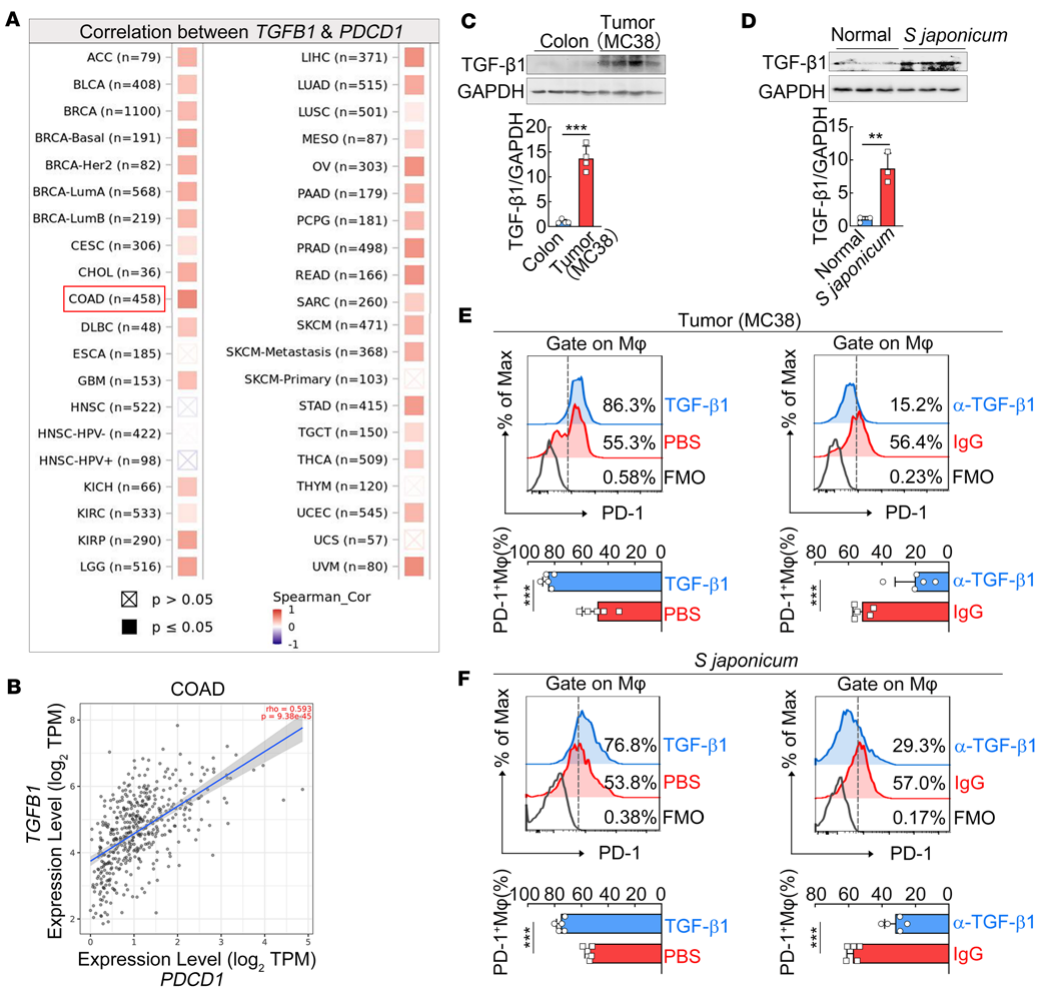
TGF-β1 is involved in PD-1 expression on macrophages in chronic inflammatory tissues. (**A**) Correlations between *PDCD1* and *TGFB1* gene expression in various human tumors by TIMER web server (TIMER2.0; https://cistrome.shinyapps.io/timer/). Abbreviations for various human tumors are given according to the database. COAD, colon adenocarcinoma. (**B**) Correlation plot between *PDCD1* and *TGFB1* gene levels in COAD (*n* = 458; Spearman correlation coefficient = 0.593, *P* = 9.38 × 10^–45^). (**C** and **D**) Immunoblot analysis of TGF-β1 expression levels in the MC38 tumors and normal colon tissues of tumor-bearing mice (**C**) or in the livers of normal uninfected and *S*. *japonicum*–infected mice (8 weeks postinfection; **D**). TGF-β1 expression levels were normalized to GAPDH. (**E** and **F**) Recombinant TGF-β1, PBS, anti–TGF-β1 neutralizing antibody, or isotype control antibody was administrated intraperitoneally into MC38 tumor–bearing (**E**) or *S*. *japonicum*–infected mice (**F**). PD-1^+^ macrophages in the tumor tissue or liver were analyzed using flow cytometry. Representative histograms and quantification of PD-1^+^ macrophages are shown. Spearman’s rank correlation coefficient (**A** and **B**) or an unpaired 2-tailed *t* test (**C**–**F**) was used for statistical analysis. The data are expressed as the mean ± SD of 3–5 mice per group and are representative of 2 independent experiments. **P < 0.01, ***P < 0.001. TPM, transcripts per million.

Abundant TGF-β1 expression is a hallmark in most pathogen- or tumor-driven chronic inflammation (25–27). The Tumor Immune Estimation Resource (TIMER) database was further employed to explore the relationship between *TGFBR1* and *PDCD1* gene expression in various human tumors. Of note, *PDCD1* gene expression was significantly positively correlated with *TGFBR1* in most types of studied human tumors, with only a few exceptions (Figure 2A). The largest correlation was observed in patients with colon cancer (*n* = 458; Figure 2B). We thus additionally selected MC38 murine colon cancer as the tumor-associated chronic inflammation model. In our animal models, immunoblot further verified higher amounts of TGF-β1 in both MC38 mouse tumors and *S*. *japonicum*–infected mouse livers (Figure 2, C and D), compared with their healthy control tissues. These data suggest the potential association between TGF-β1 and PD-1 expression in chronic inflammatory tissues.

To further establish their causative relationship, exogenous TGF-β1 and anti–TGF-β1 antibody were used in the animal models. Flow cytometry analysis revealed that TGF-β1 treatment in vivo led to a further increase of PD-1 expression on macrophages in both MC38 tumor and *S*. *japonicum*–infected mouse liver (Figure 2, E and F), while blockage of TGF-β1 using the neutralizing antibody efficiently prevented PD-1 expression on macrophages in both models (Figure 2, E and F). These results demonstrate that TGF-β1 is involved in PD-1 expression on macrophages in chronic inflammatory tissues.

### PD-1^hi^ macrophages in chronic inflammatory tissues exhibit higher expression of TGF-βRI.

To better illustrate the potential involvement of TGF-β1 signaling in inducing PD-1 expression on macrophages, we further investigated the relationship between the levels of TGF-β receptors (i.e., TGF-βRI and TGF-βRII) and PD-1. We searched public databases for *TGFBR1* and *TGFBR*2 gene expression in tumor-infiltrating immune cells. M2 macrophages also showed a preferential expression of *TGFBR1* and *TGFBR2* genes in colon, lung, and stomach adenocarcinomas based on TCGA database (Supplemental Figure 3, A and B). Further database analysis using different algorithms (TIMER2.0) revealed that *TGFBR1* gene expression displayed more significant positive correlations with infiltrating macrophages than with other infiltrating immune cell types (B cells, CD8^+^ T cells, and CD4^+^ T cells) in various human tumor types (Supplemental Figure 4). In addition, we analyzed a publicly available single-cell RNA-sequencing (scRNA-Seq) data set of human colon cancer samples (GEO accession GSE161277). Among the main infiltrating immune cell types, macrophages expressed a higher level of the *TGFBR1* gene (Supplemental Figure 5), in line with the above results obtained from TCGA database. Moreover, immunofluorescence staining of human colon cancer samples validated the presence of CD68^+^ macrophages that coexpressed TGF-βRI and PD-1 (Supplemental Figure 6).

**Figure 3:**
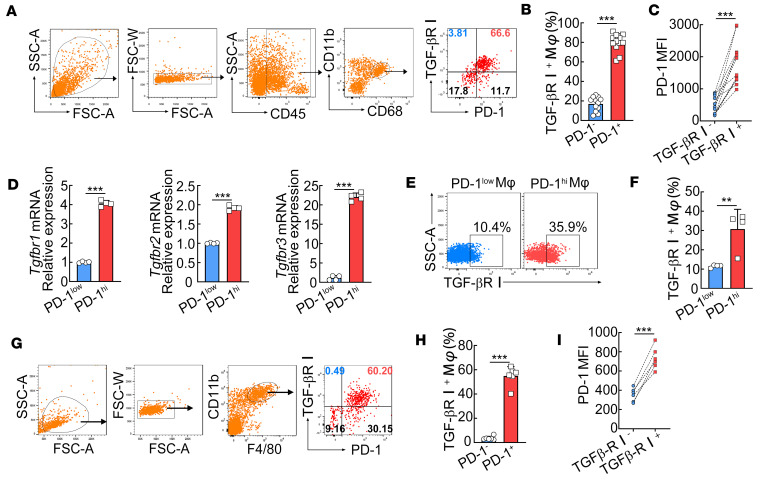
PD-1^hi^ macrophages in chronic inflammatory tissues exhibit higher expression of TGF-βRI. (**A**–**C**) PD-1 and TGF-βRI expression on macrophages in human colon cancer tissues was analyzed by flow cytometry (FCM). The gating strategy for macrophages (**A**, 4 dot plots from the left), representative dot plot (**A**, right), and quantification of TGF-βRI (**B**) and PD-1 (**C**) expression are shown (*n* = 12). (**D**–**F**) PD-1^lo^ (SiglecF^–^CD11b^+^F4/80^+^PD-1^lo^) and PD-1^hi^ macrophages (SiglecF^–^CD11b^+^F4/80^+^PD-1^hi^) were sorted from *S*. *japonicum*–infected mice (at 8 weeks postinfection) using FACS. Relative mRNA levels of *Tgfbr1*, *Tgfbr2*, and *Tgfbr3* were analyzed using real-time PCR (RT-PCR) (**D**), and protein levels of TGF-βRI were analyzed using FCM (**E** and **F**). (**G**–**I**) The gating strategy for macrophages (**G**, 3 dot plots from the left), representative FCM plot (**G**, right), the graphs of percentages showing the expression of TGF-βRI on PD-1^–^ and PD-1^+^ macrophages (**H**), and the mean fluorescence intensity (MFI) of PD-1 expression on TGF-βRI^–^ and TGF-βRI^+^ macrophages (**I**) in tumor tissues from MC38 tumor–bearing mice. An unpaired 2-tailed *t* test (**B**, **D**, **F**, and **H**) or a paired *t* test (**C** and **I**) was used for statistical analysis. The data are expressed as the mean ± SD of 4–6 mice per group and are representative of 2 independent experiments. **P < 0.01, ***P < 0.001.

To obtain a more accurate assessment, we isolated infiltrating immune cells from 12 human colon cancer samples. PD-1^+^ macrophages in colon cancer tissues expressed much higher TGF-βRI than PD-1^-^ macrophages (Figure 3, A and B). Strikingly, the vast majority of TGF-βRI^+^ macrophages (~95%) in colon cancer tissues were positive for PD-1 expression (Figure 3A), while TGF-βRI^–^ macrophages exhibited a much lower level of PD-1 expression (Figure 3, A and C). These human data suggest a potential association between enriched TGF-β1 signaling and PD-1 expression in infiltrating macrophages of chronic inflammatory tissues.

We next returned to our animal models to validate the above findings. PD-1^hi^ macrophages in *S*. *japonicum*–infected mouse livers showed significantly higher mRNA expression of *Tgfbr1*, *Tgfbr2*, and *Tgfbr3* (Figure 3D). Consistently, PD-1^hi/+^ macrophages in both *S*. *japonicum*–infected mouse livers and MC38 tumors also expressed higher TGF-βRI than PD-1^lo/–^ macrophages (Figure 3, E–H). Notably, almost all TGF-βRI^+^ macrophages (~99%) in MC38 tumors were positive for PD-1 expression, and TGF-βRI^–^ macrophages exhibited a much lower level of PD-1 expression (Figure 3, G and I). Consistent with the above results, these in vivo data also suggest that enriched TGF-β signaling is associated with higher PD-1 expression on macrophages in chronic inflammatory tissues.

### Blocking TGF-β signaling reduces PD-1 expression on macrophages in chronic inflammatory tissues.

**Figure 4:**
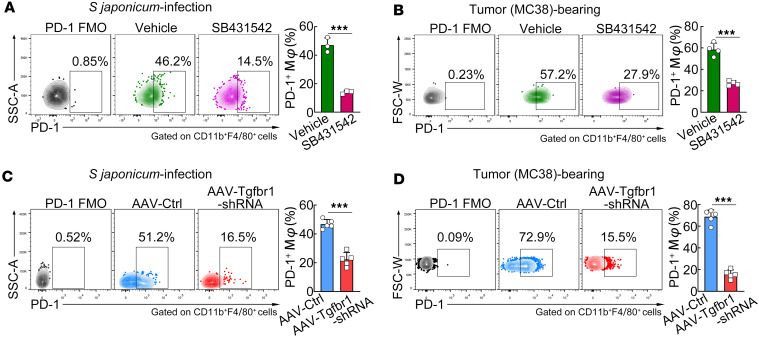
Blocking TGF-β signaling reduces PD-1 expression on macrophages in chronic inflammatory tissues. (**A** and **B**) SB431542 (TGF-βRI inhibitor) or DMSO (vehicle) was injected into *S*. *japonicum*–infected mice or MC38 tumor–bearing mice via the tail vein. (**C** and **D**) The AAV vector carrying macrophage-specific *Tgfbr1*-knockdown plasmid (AAV-F4/80-miR30-shTgfbr1) or the control vector (AAV-F4/80-miR30-Ctrl) was injected into *S*. *japonicum*–infected mice or MC38 tumor–bearing mice via the tail vein. PD-1^+^ macrophages in the liver or tumor tissue were analyzed using FCM. The representative dot plots and quantification of PD-1^+^ macrophages are shown. An unpaired 2-tailed *t* test (**A**–**D**) was used for statistical analysis. The data are expressed as the mean ± SD of 3–5 mice per group and are representative of 2 independent experiments. ***P < 0.001.

To further demonstrate whether TGF-β signaling is responsible for TGF-β–induced PD-1 expression in macrophages in chronic inflammatory tissues, MC38 tumor–bearing or *S*. *japonicum*–infected mice were treated with the TGF-βRI inhibitor SB431542. Flow cytometry analysis revealed that SB431542 treatment in mice significantly reduced PD-1 expression on macrophages, regardless of whether these macrophages were in *S*. *japonicum*–infected mouse liver (Figure 4A) or MC38 tumor (Figure 4B). To block TGF-β1 signaling more specifically, we used the adeno-associated virus (AAV) of F4/80 promoter-driven TGF-βRI knockdown (miR30-based shRNAs targeting *Tgfbr1*) to generate macrophage-specific TGF-βRI knockdown in mice in advance. The efficiency of macrophage-specific TGF-βRI knockdown was verified by FCM analysis of TGF-βRI expression on F4/80^+^CD11b^+^ peritoneal macrophages (Supplemental Figure 7, A and B), without affecting TGF-βRI expression on CD19^+^ B cells and CD3^+^ T cells (Supplemental Figure 7, A, C, and D). Flow cytometry analysis revealed that macrophage-specific TGF-βRI knockdown significantly repressed PD-1 expression on macrophages in both *S*. *japonicum*–infected mouse liver (Figure 4C) and MC38 tumor (Figure 4D). Overall, these in vivo data support that TGF-β signaling is responsible for TGF-β–induced PD-1 expression on macrophages in chronic inflammatory tissues.

### TGF-β1 directly induces PD-1 expression on macrophages in vivo and in vitro.

**Figure 5:**
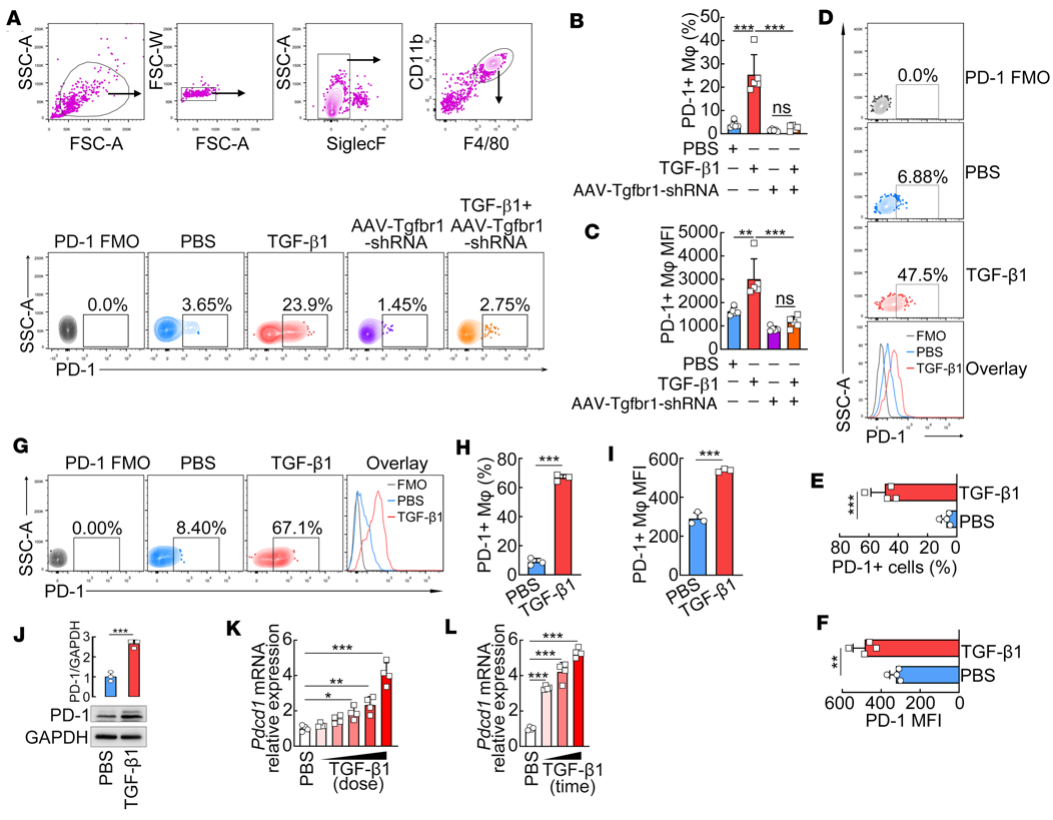
TGF-β1 induces PD-1 expression on macrophages in vivo and in vitro. (**A** and **B**) Resident peritoneal cells were collected from mice after treatment with PBS, TGF-β1, or AAV-F4/80-miR30-shTgfr1 and then analyzed for surface expression of PD-1 on CD11b^+^F4/80^+^ macrophages using FCM. The gating strategy of peritoneal macrophages (SiglecF^–^CD11b^+^F4/80^+^) is shown (**A**, upper row). The representative dot plots (**A**, bottom row) and quantification graphs of percentages (**B**) and MFI (**C**) of PD-1 expression on peritoneal macrophages are shown. (**D**–**J**) Isolated peritoneal macrophages (**D**–**F**) or RAW264.7 macrophages (**G**–**J**) were stimulated with 50 ng/mL TGF-β1 for 48 hours. FCM analysis of PD-1 expression levels was performed. The representative dot plots, histograms (**D** and **G**), and quantification graphs of percentages (**E** and **H**) and MFI (**F** and **I**) of PD-1 expression on peritoneal macrophages are shown. Immunoblot analysis of PD-1 expression was performed and the representative immunoblots are shown (**J**). (**K** and **L**) RAW264.7 macrophages were stimulated with different concentrations of TGF-β1 (0.005, 0.05, 0.5, 5, and 50 ng/mL) for 6 hours or with 50 ng/mL TGF-β1 for different times (3, 6, and 12 hours). Relative *Pdcd1* mRNA expression levels were determined using RT-PCR. A 1-way ANOVA with a Tukey’s post hoc test (**B**, **C**, **K**, and **L**) or an unpaired 2-tailed *t* test (**E**, **F**, and **H**–**J**) was used for statistical analysis. All graph data are expressed as the mean ± SD of 3–5 mice or 3 biological replicates per group and representative of 2 independent experiments. *P < 0.05, **P < 0.01, ***P < 0.001.

We next tested whether TGF-β1 induces PD-1 expression on macrophages in vivo and in vitro. Intraperitoneal injections of TGF-β1 in mice significantly increased PD-1 expression on peritoneal F4/80^+^CD11b^+^ macrophages (Figure 5, A–C). To investigate whether TGF-β1 induces PD-1 expression on macrophages by a direct action, we also used the AAV of F4/80 promoter-driven TGF-βRI knockdown to generate macrophage-specific TGF-βRI knockdown in mice. Flow cytometry analysis showed that macrophage-specific TGF-βRI knockdown in mice almost completely abrogated TGF-β1–induced PD-1 expression on F4/80^+^CD11b^+^ peritoneal macrophages (Figure 5, A–C). These results suggest that TGF-β1 directly induces PD-1 expression on macrophages in vivo.

We next isolated primary peritoneal macrophages and treated them in vitro with TGF-β1. The significant induction of PD-1 on peritoneal macrophages was also observed in vitro (Figure 5, D–F) by flow cytometry. Consistently, FCM and Western blot analyses collectively revealed that TGF-β1 in vitro stimulation also significantly increased PD-1 expression on RAW264.7 macrophages (a cell line derived from murine peritoneal macrophages; Figure 5, G–J). In addition, in vitro stimulation with TGF-β1 resulted in the upregulation of *Pdcd1* mRNA in RAW264.7 macrophages, in both a dose- and time-dependent manner (Figure 5, K and L). Together, these data demonstrate that TGF-β1 directly and sufficiently induces PD-1 expression on macrophages in vivo and in vitro.

### TGF-β1 induces PD-1 expression on macrophages independent of NFATc1.

**Figure 6:**
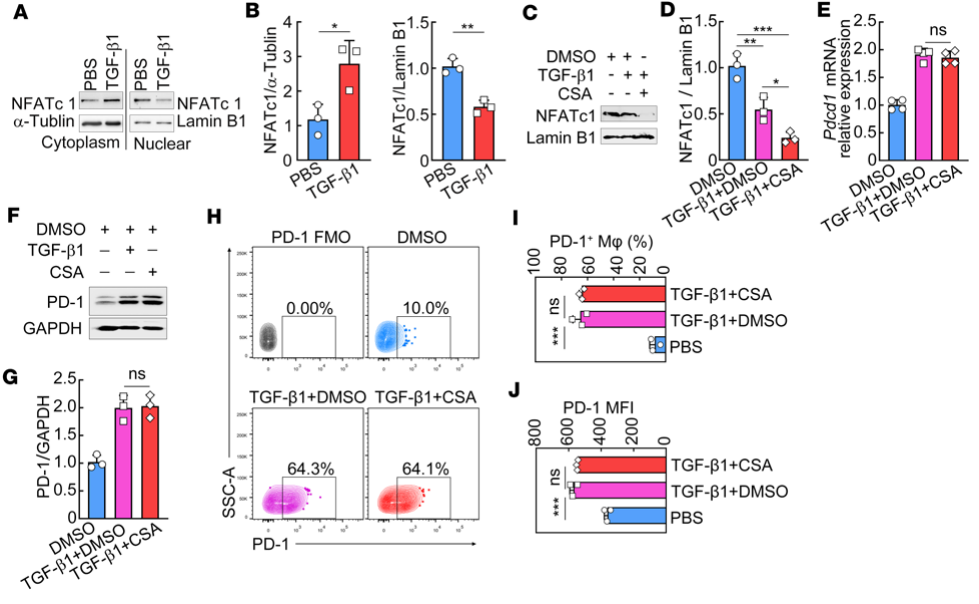
TGF-β1 induces PD-1 expression on macrophages independent of NFATc1. (**A** and **B**) RAW264.7 macrophages were stimulated with 50 ng/mL TGF-β1 for 48 hours. Cytosolic and nuclear fractions were extracted and subjected to immunoblot analysis of NFATc1. Representative immunoblots are shown (**A**). The statistical graphs show NFATc1 normalized to α-tubulin or Lamin B1. (**C**–**J**) RAW264.7 macrophages were pretreated with 1 μg/mL CsA (an inhibitor of NFATc1) or DMSO (vehicle) for an hour and then cultured with 50 ng/mL of TGF-β1 for another 3 (mRNA analysis) or 48 hours (protein analysis). Nuclear fractions were extracted and subjected to immunoblot analysis of NFATc1 (**C** and **D**). Relative *Pdcd1* mRNA expression levels were determined using RT-PCR (**E**). PD-1 protein expression levels were measured using immunoblot (**F** and **G**) and FCM (**H**–**J**). The representative immunoblots (**C** and **F**) and dot plots (**H**) are shown. The statistical graphs show NFATc1 and PD-1 normalized to GAPDH and Lamin B1, respectively (**D** and **G**). An unpaired 2-tailed *t* test (**B**) or a 1-way ANOVA with a Tukey’s post hoc test (**D**, **E**, **G**, **I**, and **J**) was used for statistical analysis. All graph data are expressed as the mean ± SD of 3 biological replicates per group and representative of 2 independent experiments. *P < 0.05, **P < 0.01, ***P < 0.001.

Given the necessary role of TCR-induced NFATc1 activity in the induction of PD-1 expression in activated CD4^+^ and CD8^+^ T cells (18, 19), we next investigated whether TGF-β1–induced PD-1 expression on macrophages requires NFATc1, though there is no TCR signaling in macrophages. Figure 6, A and B, showed significantly increased cytosolic but decreased nuclear localization of NFATc1 in TGF-β1–treated macrophages, which demonstrates that TGF-β1 treatment inhibits the nuclear translocation of NFATc1 in macrophages. Next, we also used the NFATc1 inhibitor cyclosporin A (CsA) to efficiently block the nuclear translocation of NFATc1 (Figure 6, C and D). RT-PCR (Figure 6E), immunoblot (Figure 6, F and G), and FCM (Figure 6, H–J) analyses further verified that CsA treatment did not affect TGF-β1–induced PD-1 expression in macrophages. Collectively, these results demonstrate that TGF-β1 induces PD-1 expression on macrophages independent of NFATc1 activity.

### SMAD3 and STAT3 form a complex at Pdcd1 promoter in TGF-β1–stimulated macrophages.

**Figure 7:**
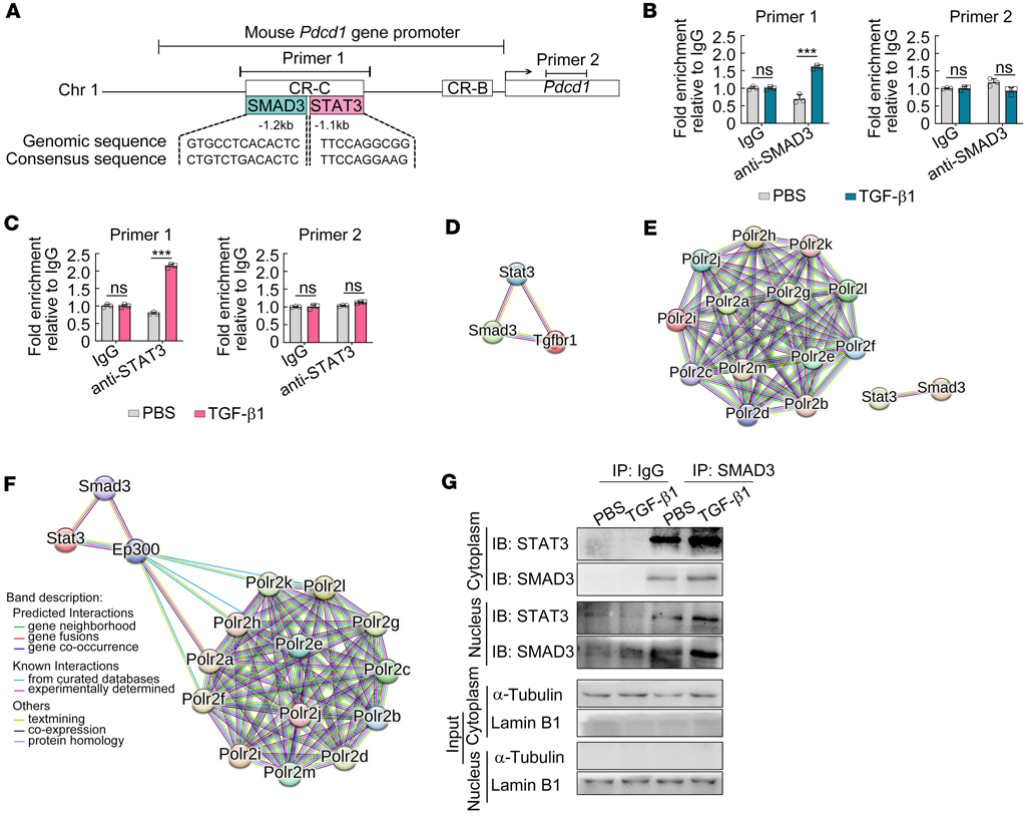
SMAD3 and STAT3 form a complex at the *Pdcd1* promoter in TGF-β1–stimulated macrophages. (**A**) Schematic diagram of mouse *Pdcd1* promoter. The cyan box and carmine box represent potential binding sites for SMAD3 and STAT3 on the promoter of the *Pdcd1* gene, respectively. Chr 1, chromosome 1; CR-B, conserved regions B; CR-C, conserved regions C. (**B** and **C**) The enrichment of SMAD3 (**B**) and STAT3 (**C**) in the *Pdcd1* promoter regions was validated by ChIP-PCR. (**D**–**F**) STRING network (https://www.string-db.org) analysis showing the interactions among proteins. (**G**) Co-immunoprecipitation (Co-IP) analysis of SMAD3 and STAT3 in nuclear and cytosolic fractions extracted from RAW264.7 macrophages with TGF-β1 or PBS treatment was performed with anti-SMAD3 or isotype IgG antibodies, followed by immunoblot analysis with anti-SMAD3 or anti-STAT3 antibody. An unpaired 2-tailed *t* test (**B** and **C**) was used for statistical analysis. All graph data are expressed as the mean ± SD of 3 biological replicates per group and are representative of 2 independent experiments. ***P < 0.001.

SMAD3 and STAT3 are known to be involved in canonical and noncanonical TGF-β signaling pathways, respectively (25, 28), and individually modulate the activity of the *Pdcd1* promoter in T cells (19, 20). However, whether SMAD3 and/or STAT3 are/is involved in promoting TGF-β1–mediated PD-1 induction in macrophages and the detailed underlying mechanism remain unclear. Two conserved regions (CR-B and CR-C), which are associated with the transcription activity of *Pdcd1* promoter, have been previously identified (17, 18). Using JASPAR, 2 putative SMAD3-binding (5′-GTGCCTCACACTC-3′) and STAT3-binding (5′-TTCCAGGCGG -3′) sites were identified and adjacently located within CR-C at 1.2 kb and 1.1 kb upstream of the transcription start site of *Pdcd1* promoter, respectively (Figure 7A). As expected, TGF-β1 stimulation significantly increased SMAD3 and STAT3 bindings to CR-C of *Pdcd1* promoter, detected by ChIP-PCR using primers that contained both adjacent putative binding sites within *Pdcd1* promoter, compared with the untreated group (Figure 7, B and C).

SMAD3 and STAT3 have been reported to activate the downstream gene transcription independently or cooperatively by direct or indirect interaction (29, 30). However, whether these 2 factors act individually or cooperatively to induce PD-1 expression and the detailed mechanism remain unclear. STRING network (https://www.string-db.org) analysis predicted direct interactions among TGF-βRI cytosolic fraction and its downstream molecules, SMAD3, and STAT3 (Figure 7D). In addition, both SMAD3 and STAT3 were predicted to have no direct interactions with RNA polymerase II (RNAP II) (Figure 7E), which is a multiprotein complex responsible for mRNA synthesis (31). However, the transcriptional coactivator p300, which can associate with both SMAD and STAT family members (32), was predicted to bridge the SMAD3/STAT3 complex and RNAP II (Figure 7F).

We then tested whether SMAD3 and STAT3 form a complex, though may not bind directly to each other, in the cytoplasm and/or in the nucleus to promote *Pdcd1* transcription. Western blot analysis following co-immunoprecipitation (Co-IP) using SMAD3 antibody revealed an increased association between SMAD3 and STAT3 in both cytosolic and nuclear fractions of macrophages with TGF-β1 stimulation (Figure 7G). These data suggest that SMAD3 and STAT3 form a complex in both cytoplasm and nucleus to promote *Pdcd1* transcription in TGF-β1–stimulated macrophages in a cooperative manner.

### TGF-β1 induces PD-1 expression on macrophages through SMAD3/STAT3 signaling.

**Figure 8:**
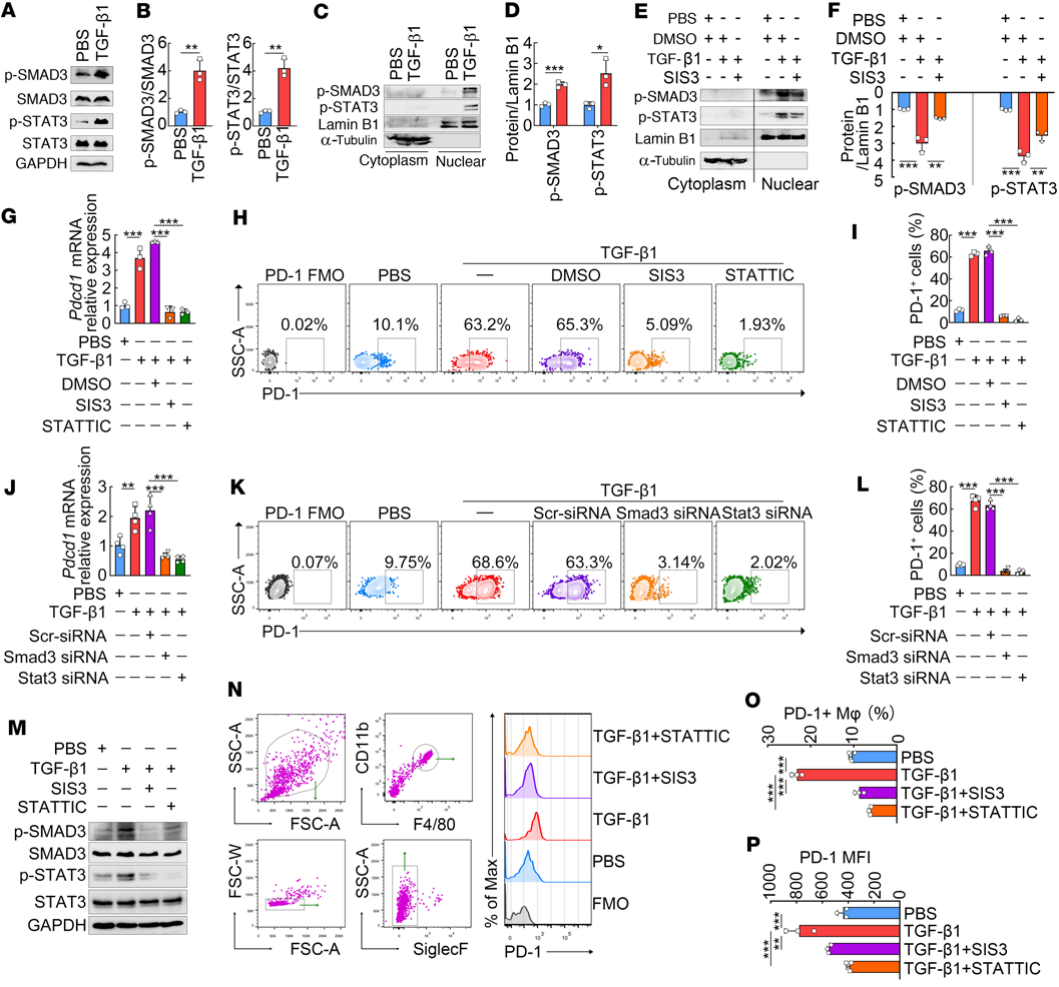
TGF-β1 induces PD-1 expression on macrophages through SMAD3/STAT3 signaling. (**A**–**D**) RAW264.7 macrophages were stimulated with 50 ng/mL TGF-β1 for an hour. Immunoblot analysis of p-SMAD3 and p-STAT3 in whole-cell lysates (**A** and **B**) and nuclear fractions (**C** and **D**). (**E** and **F**) RAW264.7 macrophages were pretreated with 10 nmol/mL SIS3 (p-SMAD3 inhibitor) or DMSO (vehicle) for an hour and then stimulated with 50 ng/mL of TGF-β1 for another hour. Immunoblot analysis of p-SMAD3 and p-STAT3 in cytosolic and nuclear extracts. (**G**–**I**) RAW264.7 macrophages were pretreated with 10 nmol/mL SIS3 (p-SMAD3 inhibitor), 10 nmol/mL Stattic (p-STAT3 inhibitor), or DMSO (vehicle) for an hour, then cultured with 50 ng/mL TGF-β1 for another 3 hours (mRNA analysis) or 24 hours (protein analysis). RT-PCR (**G**) and FCM analysis (**H** and **I**) were used for assessing mRNA and protein expression, respectively. (**J**–**L**) RAW264.7 macrophages were transfected with scrambled (Scr) siRNA, Smad3-specific siRNA, or STAT3-specific siRNA, then cultured with 50 ng/mL TGF-β1 for another 3 hours (mRNA analysis) or 24 hours (protein analysis). RT-PCR (**J**) and FCM analysis (**K** and **L**) were used for assessing mRNA and protein expression, respectively. (**M**–**P**) C57BL/6 mice were intraperitoneally injected with TGF-β1 (0.5 μg per mouse), SIS3 (50 μg/mouse), or Stattic (75 μg/mouse) for 5 consecutive days. Peritoneal macrophages were collected for immunoblot analysis of p-SMAD3 and p-STAT3 (**M**) and FCM analysis of PD-1 (**N**–**P**). The representative dot plots (**N**) and quantification graphs of percentages (**O**) and MFI (**P**) of PD-1 expression on peritoneal macrophages are shown. An unpaired 2-tailed *t* test (**B** and **D**), or a 1-way ANOVA with Tukey’s post hoc test (**F**, **G**, **I**, **J**, **L**, **O**, and **P**), was used for statistical analysis. The data are expressed as the mean ± SD of 3 mice or 3 biological replicates per group. *P < 0.05, **P < 0.01, ***P < 0.001.

We then determined whether TGF-β signaling could drive the activation and nuclear translocation of SMAD3 and STAT3 in macrophages. In response to TGF-β1, phosphorylated SMAD3 (S423/425, p-SMAD3) and STAT3 (Y705, p-STAT3) levels were increased but without alteration of the total SMAD3 and STAT3 levels in macrophages (Figure 8, A and B). Western blot analysis of nuclear lysates further demonstrated that TGF-β1 induced nuclear translocation of p-SMAD3 and p-STAT3 in macrophages (Figure 8, C and D). To further investigate the relationship between SMAD3 and STAT3, we utilized a specific p-SMAD3 inhibitor, SIS3, to inhibit SMAD3 phosphorylation. In vitro treatment with SIS3 not only inhibited TGF-β1–induced SMAD3 phosphorylation but also reduced the phosphorylation of STAT3 (Figure 8, E and F), which has been reported to be activated by upstream SMAD3 to activate transcription of Snail and to lead to β cell dysfunction (33).

We next applied specific p-SMAD3 and p-STAT3 inhibitors (SIS3 and Stattic, respectively) to address whether SMAD3 and STAT3 are responsible for TGF-β1–induced PD-1 expression on macrophages. In vitro treatment with either SIS3 or Stattic almost entirely abrogated TGF-β1–induced PD-1 mRNA and protein expression in macrophages, as assessed by a combination of RT-PCR and FCM analyses (Figure 8, G–I). The roles of SMAD3 and STAT3 were also verified through a siRNA knockdown experiment. The siRNA efficiencies were verified by RT-PCR (Supplemental Figure 8). As expected, basically consistent results were obtained using SMAD3 and STAT3 siRNAs (Figure 8, J–L). Next, we investigated the in vivo treatment effects of p-SMAD3 and p-STAT3 inhibitors in TGF-β1–injected mice. Consistent with in vitro results, immunoblot showed that in vivo treatment with SIS3 inhibited both SMAD3 and STAT3 phosphorylation in peritoneal macrophages of TGF-β1–injected mice (Figure 8M). As indicated by FCM analysis, following intraperitoneal injections of TGF-β1, both the percentage and MFI of PD-1 expression on peritoneal macrophages were significantly increased (Figure 8, N–P). However, treatment with either SIS3 or Stattic in mice also completely abrogated TGF-β1–induced PD-1 expression on peritoneal macrophages (Figure 8, N–P). Together, our in vitro and in vivo experiments collectively demonstrate that TGF-β1 induces PD-1 expression on macrophages through cooperative signaling of SMAD3/STAT3.

## Discussion

Macrophages play critical roles in regulating tissue homeostasis and inflammation and display remarkable plasticity, changing their phenotypes and functions based on their local tissue milieu (21, 34, 35). PD-1 expression is a global mechanism for suppressing immune responses across innate and adaptive cells (2). However, PD-1 expression is not unique to T cells. It is also highly and permanently expressed on macrophages in a variety of chronic infections or tumors (hepatitis C virus infection, pneumocystis pneumonia, gastric cancer, colorectal cancer, etc.) (11, 13–15, 36), which have received growing attention recently for the essential role in dampening immunity, further emphasizing the need for the mechanistic elucidation of PD-1 expression in macrophages. Notably, our analysis based on databases found that infiltrating macrophages in common human tumors possess a higher expression of the *PDCD1* gene than other infiltrates. However, it is currently unclear how a chronic inflammatory environment enables macrophages to sustain high expression of PD-1, although such insight is essential to optimize future PD-1–targeting therapeutics. Herein, we have shown that macrophages adapt to their microenvironment cue, TGF-β1, to induce PD-1 expression, representing a special aspect of TGF-β1–mediated regulation of tissue-specific immunity.

Notably, myeloid cell–specific PD-1 ablation led to more effective inhibition of tumor growth compared with T cell–specific PD-1 ablation (4–6). Macrophages integrate tissue- and inflammation-specific cues from the microenvironment that can enhance or suppress local immune responses (35). During chronic infections, extensively increased PD-1 expression has been found on macrophages (23). Although PD-1 function is well confirmed to regulate T cell function, PD-1–conferred unique immune signatures of macrophages in inflamed tissues during chronic infections may not be the same as those of T cells and remain incompletely characterized. Of note, a recent study provided the first evidence that PD-1^+^ and PD-1^–^ macrophages in tumor tissues possess distinct transcriptomic profiles, suggesting the role of PD-1 in restraining the differentiation, activation, and costimulatory function of macrophages that suppresses antitumor immunity (6). Interestingly, our study, from the perspective of chronic infection, not only showed extensively increased PD-1 expression on macrophages in chronically inflamed livers with chronic infection but also revealed transcriptome diversity between PD-1^hi^ and PD-1^lo^ macrophages by RNA-Seq analysis. In addition, our study further validated by FCM that PD-1–deficient macrophages in *S*. *japonicum*–infected mice had a diminished expression of other immune-inhibitory molecules (CTLA-4, PD-L1, and PD-L2) that convey immunosuppressive functions. Although the exact underlying mechanism remains unclear and is not the main focus of this study, these data cooperatively provide clues about PD-1–dominated changes in immunomodulatory features of macrophages for dampening immune responses in inflamed tissues with chronic infection or tumor, which may be worth exploring further.

Upon initial immune stimulus at the acute inflammation stage, PD-1 is transiently expressed on both innate and adaptive immune cells (9, 17). During chronic immune stimulation, PD-1 remains highly and permanently sustained on a variety of immune cells, leading to their functional suppression (23). Emerging evidence supports the notion that PD-1 expression is environmental context dependent (2). TGF-β1 is a master immunoregulator and is overproduced in tolerogenic environments of chronic infections or tumors (25–27). Importantly, by surveying public databases, we observed significant positive correlations between *PDCD1* and *TGFB1* gene expression in most human tumor types. Studies have shown that multiple cytokines, including TGF-β1, participate in the regulation of TCR-stimulated PD-1 expression on T cells (20). However, our study revealed the strong capacity of TGF-β1 to sufficiently and independently induce PD-1 expression on macrophages. Of note, TGF-β1–induced PD-1 expression on macrophages is independent of its activation status and highly context dependent, which is different from its induction on T cells. In addition to TGF-β1 level, we found that almost all TGF-βRI^+^ macrophages in these chronic inflammatory tissues were positive for PD-1 expression, and TGF-βRI^–^ macrophages expressed a much lower level of PD-1. Thus, these observations also emphasize the functional plasticity of macrophage phenotype is tightly congruent with the local microenvironment.

TCR signaling–triggered activation of NFATc1 was previously shown to be required for initiating PD-1 expression in T cells (17–19). Meanwhile, TGF-β1 promotes NFATc1 binding on the *Pdcd1* promoter in T cells and enhances PD-1 expression (20, 37). Intriguingly, we found that in macrophages, important innate immune cells without TCR expression, TGF-β1 itself sufficiently and directly induced and sustained higher PD-1 expression under antiinflammatory conditions, independent of NFATc1 activity. These results may demonstrate that fundamental mechanisms governing PD-1 expression are distinct across different cell types and that macrophages can utilize various microenvironmental signals and cell pathways to initiate or sustain PD-1 expression.

Our data showed that inhibition of SMAD3 or STAT3 completely abrogated TGF-β1–induced PD-1 expression on macrophages, suggesting a potential interplay between SMAD3 and STAT3 on TGF-β1 stimulation in macrophages. Existing studies have reported a TGF-β1–mediated association between SMAD3 and STAT3 in the regulation of hepatic or cardiac cell fibrogenesis, tumor cell fibrogenesis, and β cell dysfunction, cooperating with or antagonizing each other (28–30, 33, 38, 39). However, the detailed mechanisms of SMAD3 and STAT3 in regulating PD-1 expression of macrophages remain unclear. Here, we revealed a noncanonical TGF-β1/SMAD3/STAT3 axis in macrophages by which SMAD3 and STAT3 physically interacted with each other in the cytoplasm and translocated into the nucleus of macrophages to induce PD-1 expression in macrophages. Studies suggest that STAT3 can be activated by a number of additional local signals (e.g., IL-10, IL-6, EGF) (40). It is reasonable to postulate synergistic coactivators may exist to regulate PD-1 induction in macrophages. Increasing evidence indicates that SMAD3 and STAT3 engage in crosstalk in a highly context-dependent manner (30). Considering the crucial roles of PD-1 on macrophages in tolerogenic local environments, further studies are intensively needed to elucidate the divergent, context-dependent SMAD3/STAT3 cooperation for PD-1 induction in macrophages.

In summary, our data provide both clinical relevance and experimental evidence to support a critical role of TGF-β1 in maintaining the high level of PD-1 expression on macrophages in chronic inflammatory tissues through cooperative signaling of SMAD3/STAT3. The mechanistic understanding offers the prospect of targeting PD-1 to intervene with or augment immune responses in chronic disorders, including chronic infections and cancers.

## Methods

### Sex as a biological variable.

Sex was not considered as a biological variable. In the early stage of this study, we examined male and female mice, and similar findings were observed in both sexes.

### Database analysis.

Correlation analysis for gene expression in tumors was performed using GEPIA (http://gepia2021.cancer-pku.cn/index.html), a web-based tool with high-throughput RNA-Seq data (TCGA and GTEx databases) (41). The TIMER2.0 web server (https://cistrome.shinyapps.io/timer/) was used for investigating the correlations between genes and immune infiltrates in various tumor types (42, 43). The correlation was evaluated by the Spearman correlation test. *P* values less than 0.05 were considered statistically significant.

### Isolation of human tumor-infiltrating immune cells.

Human tumor-infiltrating immune cells were isolated as previously described (44, 45). Briefly, fresh human colon cancer samples were washed in PBS and then minced mechanically into small pieces. The tissue pieces were then enzymatically digested with 4 mg/mL collagenase IV (MilliporeSigma) and 80 U/mL DNase I (MilliporeSigma) in RPMI 1640 medium (Gibco, Thermo Fisher Scientific) containing 4% FBS for 60 minutes in a 37°C shaking water bath. The digested tissues were filtered through a 40 μm cell strainer (Yanjin). The isolated cells were washed with PBS, resuspended in 7 mL 20% Percoll (GE Healthcare, now Cytiva), and overlaid on 2 mL of 40% Percoll in a 15 mL tube. Percoll gradient separation was performed by centrifugation at 500*g* for 30 minutes at 4°C. Infiltrating immune cells were collected at the Percoll gradient interface and washed with PBS containing 2% FBS.

### Mice.

Eight-week-old male C57BL/6 mice were housed under specific pathogen–free conditions at the Laboratory Animal Center of Nanjing Medical University (Nanjing, China). *Pdcd1*^–/–^ mice (B6/129S6) were provided by Wenyue Xu (Army Medical University, Chongqing, China). All mice were bred and maintained in a temperature- and humidity-controlled animal facility with a 12-hour light/12-hour dark cycle.

### S. japonicum infection of mice.

Mice were exposed percutaneously to 12 ± 1 *S*. *japonicum* cercariae (Chinese mainland strain) shed from infected *Oncomelania hupensis* snails, which were obtained from the National Institute of Parasitic Diseases, Chinese Center for Disease Control and Prevention, Shanghai, China.

### Tumor implantation.

The subcutaneous tumor–bearing mouse model was established by subcutaneously injecting 5 × 10^6^ MC38 cells into the right hind limbs of C57BL/6 mice as previously described (46).

### Recombinant cytokine, neutralizing antibody, or inhibitor treatment in mice.

Recombinant TGF-β1 (BioLegend; 0.5 μg per mouse) was administrated intraperitoneally into normal mice daily for 5 consecutive days. For *S*. *japonicum*–infected or tumor-bearing mice, recombinant TGF-β1 was administrated intraperitoneally every 2 days, starting at 6 weeks postinfection until 8 weeks postinfection or starting at 7 days after tumor implantation until 21 days postimplantation.

Anti–TGF-β1 neutralizing antibody (BE0057; BioXCell; 250 μg per mouse) was administrated intraperitoneally into mice daily every 3 days, starting at 6 weeks postinfection until 8 weeks postinfection or starting at 7 days after tumor implantation until 21 days postimplantation.

For in vivo SMAD3 or STAT3 activity inhibition, chemical inhibitor SIS3 (Selleck; 50 μg per mouse) or Stattic (Selleck; 75 μg per mouse) was administrated intraperitoneally into mice 1 hour before each TGF-β1 injection.

At 6 weeks after *S*. *japonicum* infection or on day 7 after tumor implantation, mice were injected with SB431542 (10 mg/kg per mouse), an inhibitor of the tyrosine kinase activity of TGF-βRI, via the tail vein every other day for a total of 7 times.

All the mice were sacrificed 2 days after the last injection.

### Recombinant AAV and macrophage-targeting interference with specific promoter.

An AAV serotype 2/9 vector carrying macrophage-specific TGF-βRI–knockdown plasmid (AAV-F4/80-miR30-shTgfbr1) and the corresponding control vector (AAV-F4/80-miR30-Ctrl) expressing scrambled shRNA were obtained as ready-to-use viral stock from Hanbio Biotechnology. The AAV2/9 system harbors an macrophage-specific synthetic promoter F4/80 (47), a miR30-based shRNA targeting *Tgfbr1*, a cytomegalovirus promoter, and an enhanced GFP reporter. The nucleotide shRNA of *Tgfbr1* was cloned using the following sequence: 5′-GCTGACAGCTTTGCGAATTAA-3′. Mice were injected with 10^11^ viral particles via the tail vein 1 month before intraperitoneal injection of TGF-β1, *S*. *japonicum* infection, or tumor implantation.

### In vitro siRNA transfection.

SMAD3 and STAT3 knockdown in RAW264.7 macrophages were achieved by specific siRNA transfection. The siRNAs specific for mouse *Smad3* and *Stat3* and the scrambled siRNA were purchased from GenePharma. Transfection of siRNA was performed using Lipofectamine 2000 reagent (Invitrogen) according to the manufacturer’s instructions. The silencing efficiency was assayed by RT-PCR. Following transfection, cells were cultured as described below.

### Purification of peritoneal macrophages and cell culture.

Peritoneal macrophages were collected from the peritoneum as described previously (48). Briefly, resident peritoneal cells were harvested from mice by peritoneal lavage. Peritoneal exudate cells were allowed to adhere for 2 hours. Then, nonadherent cells were removed to achieve more than 90% purity of macrophages.

Murine RAW264.7 macrophages were obtained from the Cell Bank of Chinese Academy of Science (Shanghai, China). RAW264.7 macrophages or purified peritoneal macrophages were maintained in DMEM (Gibco) supplemented with 10% (v/v) FBS (Gibco) and 1% (v/v) antibiotics (penicillin/streptomycin; Gibco).

To investigate the impact of TGF-β1 on PD-1 expression, macrophages were cultured for 3 (mRNA analysis) or 48 (protein analysis) hours in the presence of TGF-β1 (50 ng/mL) as previously described (49).

For inhibitor treatment, macrophages were pretreated with CsA (NFATc1 inhibitor, 1 μg/mL), Stattic (10 nmol/mL), or SIS3(10 nmol/mL) for an hour, respectively (all inhibitors from Selleck), then cultured with 50 ng/mL of TGF-β1 for another 3 (mRNA analysis) or 48 (protein analysis) hours. For detection of the phosphorylation of SMAD3 or STAT3, macrophages were stimulated with TGF-β1 (50 ng/mL) for an hour.

### Isolation of PD-1^lo^ and PD-1^hi^ macrophages.

At 8 weeks after *S*. *japonicum* infection, the mouse livers were perfused and digested using the collagenase method, followed by Percoll density gradient centrifugation to isolate hepatic mononuclear cells as described previously (50). The cells were stained with the following fluorescence-conjugated antibodies: anti-SiglecF (clone E50-2440; BD Biosciences), anti-CD11b (clone M1/70; BD Biosciences), anti-F4/80 (clone BM8; eBioscience), and anti–PD-1 (clone J43; eBioscience). The PD-1^lo^ macrophages (SiglecF^–^CD11b^+^F4/80^+^PD-1^lo^) and PD-1^hi^ macrophages (SiglecF^–^CD11b^+^F4/80^+^ PD-1^hi^) were analyzed and sorted using a FACSAria II cell sorter (BD Biosciences).

### RNA-Seq and bioinformatic analysis.

Total RNA samples from isolated PD-1^lo^ or PD-1^hi^ macrophages were extracted using TRIzol Reagent (Invitrogen) and subjected to RNA-Seq analysis by BGI company (Shenzhen, China). GO and KEGG pathway enrichment analyses were performed using the BGI service online platform.

### Western blot.

Cell or tissue samples were lysed using RIPA Lysis and Extraction Buffer (Thermo Fisher Scientific) containing a protease inhibitor cocktail (Thermo Fisher Scientific). Cytosolic and nuclear proteins were extracted using NE-PER Nuclear and Cytoplasmic Extraction Reagents (Thermo Fisher Scientific) according to the manufacturer’s instructions. Protein concentrations in the extracts were measured using a Pierce BCA Protein Assay Kit (Thermo Fisher Scientific). The proteins were then denatured, separated by 12% SDS-PAGE, and blotted onto PVDF membranes (Bio-Rad). The blotted membranes were blocked with 5% skim milk in phosphate-buffered saline containing 0.1% Tween-20 (PBS-T) for 1 hour at room temperature and then incubated with primary antibodies overnight at 4°C. Following washing with PBS-T, the membranes were incubated with HRP-conjugated secondary antibodies for 1 hour at room temperature. Immunoblots were visualized using a ChemiDoc MP Imaging System (Bio-Rad). Relative protein expression levels were quantified by densitometric analysis using ImageJ software (NIH; http://imagej.nih.gov/ij).

The following primary and secondary antibodies were used in this study: anti–TGF-β1 (ab179695; Abcam), anti–PD-1 (PA5-20351; Invitrogen), anti-GAPDH (5174; Cell Signaling Technology [CST]), anti-NFATc1 (8032; CST), anti–α-tubulin (2148; CST), anti–Lamin B1 (13435; CST), anti–p-STAT3 (9145; CST), anti-STAT3 (12640; CST), anti–p-SMAD3 (9520; CST), anti-SMAD3 (9523; CST), and HRP-conjugated anti-rabbit IgG (7074; CST).

### Quantitative RT-PCR.

The total RNA of tissues or cells was extracted and quantified using TRIzol Reagent (Invitrogen) or NanoDrop spectrophotometer (Thermo Fisher Scientific), respectively. Isolated RNA (1 μg) was then reverse-transcribed to generate cDNA using 5× All-In-One RT MasterMix (ABM) according to the manufacturer’s instructions. RT-PCR was performed with the PowerUp SYBR Green Master Mix kit (Applied Biosystems) on a StepOnePlus Real-Time PCR System instrument (Applied Biosystems). Relative gene expression levels were normalized against the expression of *Actb* gene (encoding β-actin) and expressed as fold-change compared with the control. The following primers were used in this study: *Pdcd1*, forward, 5′-ACCCTGGTCATTCACTTGGG-3′, and reverse, 5′-CATTTGCTCCCTCTGACACTG-3′; *Arg1*, forward, 5′-CTCCAAGCCAAAGTCCTTAGAG-3′, and reverse, 5′-AGGAGCTGTCATTAGGGACATC-3′; *Cd274* (encoding PD-L1), forward, 5′-GCTCCAAAGGACTTGTACGTG-3′, and reverse, 5′-TGATCTGAAGGGCAGCATTTC-3′; *Pdcd1lg2* (encoding PD-L2), forward, 5′-CTGCCGATACTGAACCTGAGC-3′, and reverse, 5′-GCGGTCAAAATCGCACTCC-3′; *Tgfb1*, forward, 5′-TGACGTCACTGGAGTTGTACGG-3′, and reverse, 5′-GGTTCATGTCATGGATGGTGC-3′; *Il10*, forward, 5′-GCTCTTACTGACTGGCATGAG-3′, and reverse, 5′-CGCAGCTCTAGGAGCATGTG-3′; *Tgfbr1*, forward, 5′-TCTGCATTGCACTTATGCTGA-3′, and reverse, 5′-AAAGGGCGATCTAGTGATGGA-3′; *Tgfbr2*, forward, 5′-CCGCTGCATATCGTCCTGTG-3′, and reverse, 5′-AGTGGATGGATGGTCCTATTACA-3′; *Tgfbr3*, forward, 5′-GGTGTGAACTGTCACCGATCA-3′, and reverse, 5′-GTTTAGGATGTGAACCTCCCTTG-3′; *Actb*, forward, 5′-GGCTGTATTCCCCTCCATCG-3′, and reverse, 5′-CCAGTTGGTAACAATGCCATGT-3′.

### FCM analysis.

Following collagenase digestion of tumor and liver homogenate and filtration through a cell strainer, mononuclear cells were isolated by gradient centrifugation with Percoll as previously described (50, 51). In addition, resident peritoneal cells were harvested from mice by peritoneal lavage. Before surface staining, all cell samples were preincubated with anti-CD16/32 (clone 93; Thermo Fisher Scientific) to block Fc receptors at 4°C for 15 minutes. Then cells were stained with indicated fluorescently labeled antibodies for 30 minutes at 4°C in the dark. The following antibodies were used for surface staining: anti-mouse SiglecF (clone E50-2440; BD Biosciences), anti-mouse/human CD11b (clone M1/70; eBioscience), anti-mouse F4/80 (clone BM8; eBioscience), anti-mouse PD-1 (clone J43; eBioscience), anti-mouse CTLA-4 (clone UC10-4B9, Invitrogen), anti-mouse PD-L1 (clone MIH5, Invitrogen), anti-mouse PD-L2 (clone TY25, Invitrogen), anti-human CD45 (clone HI30; eBioscience), anti-human CD68 (clone Y1/82A; BioLegend), anti-human PD-1 (clone MIH4; Invitrogen), and anti-human TGF-βRI (clone 141231; R&D Systems). Following immunofluorescence staining, cells were analyzed using a FACSVerse (BD Biosciences). All FCM data were analyzed by FlowJo software (Version 9; Tree Star).

### ChIP.

The ChIP experiment was performed using the SimpleChIP Enzymatic Chromatin IP Kit (9002; CST) according to the manufacturer’s instructions. Briefly, macrophages were treated with TGF-β1 (50 ng/mL) for 3 hours. For an individual ChIP assay, a total of 2 × 10^6^ cells were fixed with 1% formaldehyde for 15 minutes at room temperature for cross-linking the proteins to DNA, followed by 5 minutes of incubation with 0.125 M glycine to stop the fixation. Then the isolated chromatin DNA was sonicated and sheared to lengths between 200 bp and 900 bp. Chromatin DNA shearing efficiency was evaluated by agarose gel electrophoresis. The sheared DNA was immunoprecipitated at 4°C overnight with anti-SMAD3 (9523; CST) or anti-STAT3 antibody (12640; CST) or a homologous IgG (2729; CST). The co-precipitated DNA samples were purified and then subjected to RT-PCR. The following primers targeting *Pdcd1* promoter were used: primer 1, sense 5′-CCTAGCTTCTGCCCACAGG-3′, and antisense 5′-TCTCTGTGTTTCGCCACAGT-3′; primer 2, sense 5′-CAGAGGCCACTCTTGACTCC-3′, and antisense 5′-AAGGCTCCCTGGAGGAGATA-3′.

### Immunoprecipitation and immunoblotting.

Macrophages treated with TGF-β1 were lysed in RIPA Lysis Buffer supplemented with a protease inhibitor cocktail (Thermo Fisher Scientific). Cytosolic and nuclear proteins were extracted as described above. Pierce BCA Protein Assay Kit (Thermo Fisher Scientific) was used to measure protein concentration. For immunoprecipitation, 500 μg of cytosolic or nuclear proteins was incubated overnight at 4°C with anti-SMAD3 (9523; CST) or a homologous IgG (2729; CST), then immunoprecipitated with Protein A/G agarose beads (37478; CST) for 2 hours at 4°C. Formed immune complex was spun down, washed 3 times with co-immunoprecipitation buffer, solubilized with sample loading buffer, and resolved by SDS-PAGE. Input cell lysates and immunoprecipitate were analyzed by Western blot as described above.

### Statistics.

Statistical analysis was performed using the SPSS software (Version 26; IBM), and the data are presented as the mean ± SD. The comparisons between 2 groups were evaluated with the 2-tailed *t* test, while the comparison of multiple groups was evaluated by using a 1-way ANOVA, followed by Tukey’s post hoc test. *P* < 0.05 was considered significant.

### Study approval.

All animal experimental procedures were approved by the Institutional Animal Care and Use Committee (IACUC) of Nanjing Medical University (Permit IACUC-1804025). All experiments involving animals were performed in accordance with the Regulations for the Administration of Affairs Concerning Experimental Animals. All efforts were made to minimize animal suffering for all procedures and to reduce the number of animals used.

Human colon carcinoma samples were obtained from 12 patients with colon cancer who underwent routine surgery and pathological examination. Patients’ written informed consents were obtained. The study protocol was approved by the Ethics Committee of Nanjing Medical University (No. 2022-942).

### Data availability.

The raw RNA-Seq data have been deposited in the NCBI Sequence Read Archive (https://www.ncbi.nlm.nih.gov/sra/) under accession number PRJNA827953. scRNA-Seq data are publicly available as a GEO data set (GSE161277). All raw data values for graphs are provided in the Supporting Data Values file. Any additional information is available from the corresponding authors upon reasonable request.

## Author contributions

CS, SZ, RT, ZL, YW, XC, and XY designed the study. ZL, RT, YW, CM, WX, JS, JY, XW, and XQ performed the experiments. ZL, RT, YW, SZ, CS, XC, and JZ analyzed and interpreted the data. CW, LX, XC, JZ, YL, XZ, CY, and XY provided technical or material support. CS and SZ supervised the project. ZL, SZ, and CS wrote and/or revised the paper.

## Supplementary Material

Supplemental data

Unedited blot and gel images

Supporting data values

## Version 1 03/05/2024

In-Press Preview

## Version 2 04/08/2024

Electronic publication
