# Supplementary material for: TGF-β1 induces PD-1 expression in macrophages through SMAD3/STAT3 cooperative signaling in chronic inflammation
Source: JCI Insight. 2024 Mar 5;9(7):e165544. doi: 10.1172/jci.insight.165544 (PMC11128204; doi:10.1172/jci.insight.165544)
Supplement: Supplemental data [file jciinsight-9-165544-s170.pdf]

Supplementary Figures and Figure Legends

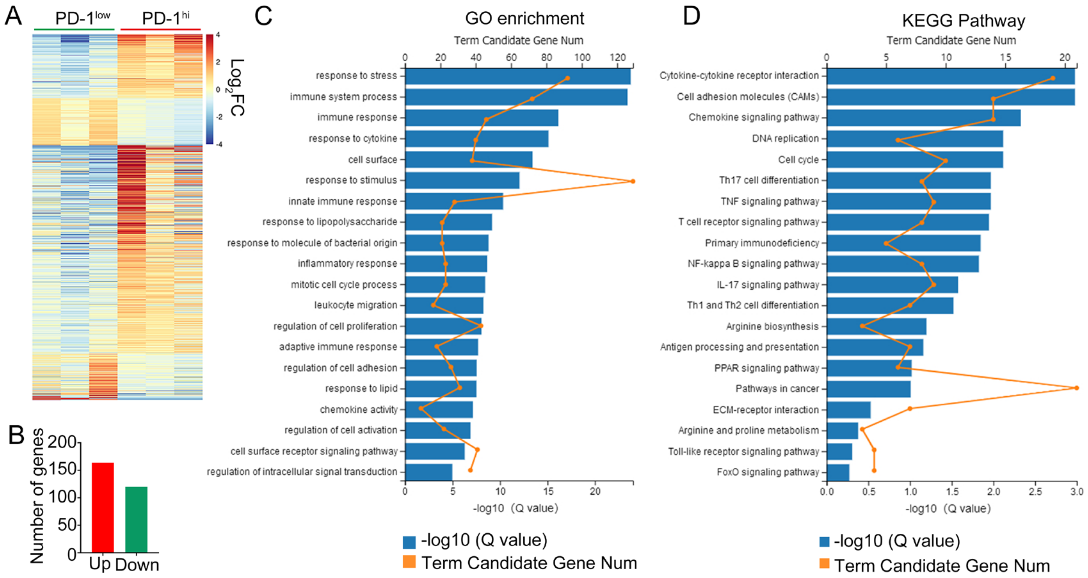

Figure S1. RNA sequencing of PD-1^{low} Mφ and PD-1^{hi} Mφ in chronic inflammatory tissues. (**A**) Heatmap showing a total of 941 differentially expressed genes (DEGs) between PD-1^{low} and PD-1^{hi} Mφ. (**B**) Among the 284 DEGs with equal or greater than 2-fold change, 164 were up-regulated (red column) and 120 were down-regulated (green column) in PD-1^{hi} Mφ. (**C** and **D**) GO (**C**) and KEGG pathway (**D**) analyses of 941 DEGs between PD-1^{low} and PD-1^{hi} Mφ.

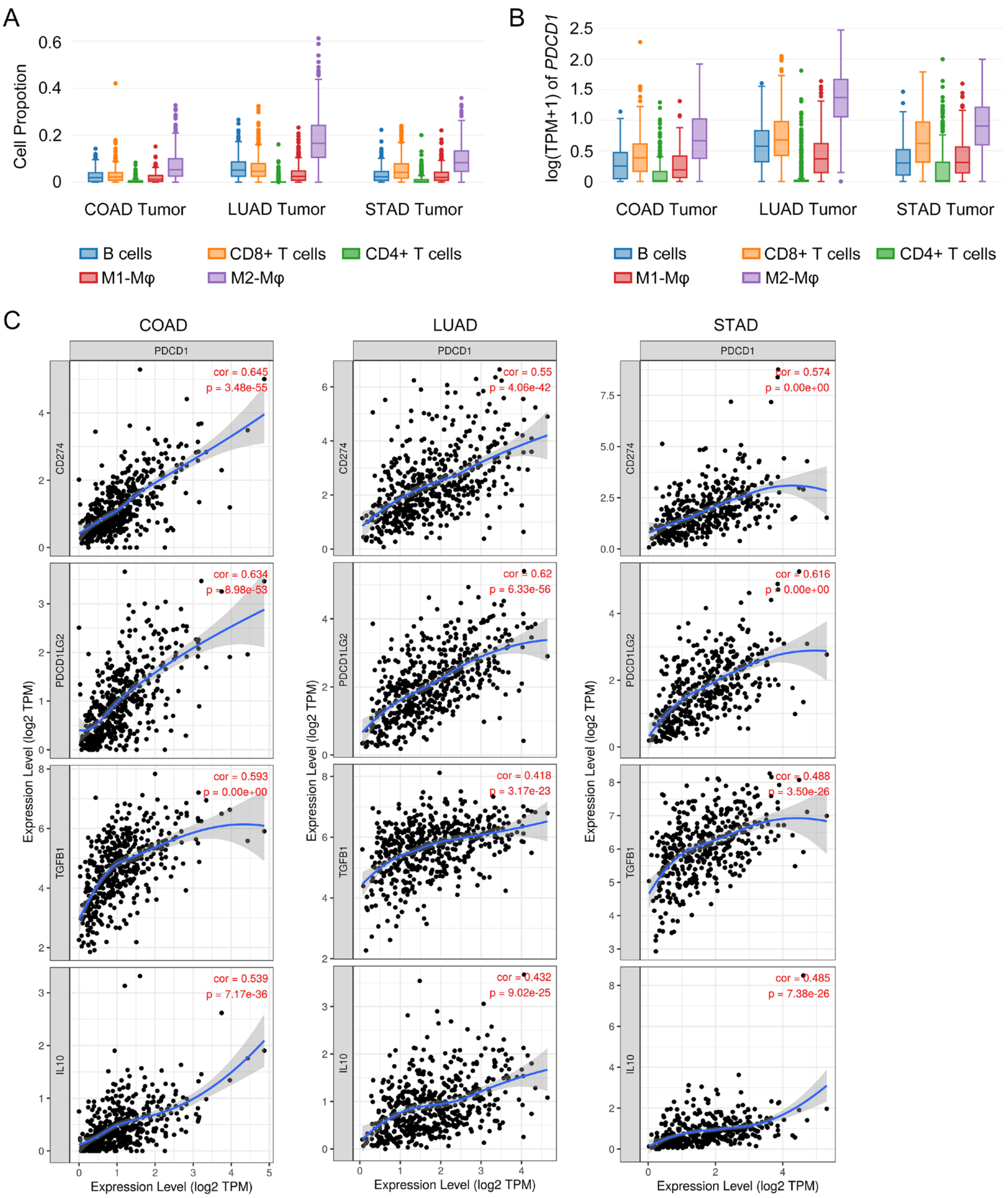

Figure S2. ***PDCD1*** **is preferentially expressed by infiltrating M2-Mφ and is positively correlated with immunosuppressive genes.** (**A** and **B**) The proportion of infiltrating immune cells (**A**) and their *PDCD1* gene expression (**B**) in common human tumors (COAD, colon adenocarcinoma; LUAD, lung adenocarcinoma; STAD, stomach adenocarcinoma; based on GEPIA platform, http://gepia2021.cancer-pku.cn/index.html). (**C** and **D**) Heat map (**C**) showing the correlations between *Pdcd1* and immunosuppressive genes (*CD274*, *PDCD1LG2*, *TGFB1*, and *IL10*) in various human tumors. Representative correlation plots (**D**) between *Pdcd1* and immunosuppressive genes in COAD (n=458), LUAD (n=515), and STAD (n=415). The TIMER2.0 web server (https://cistrome.shinyapps.io/timer/) was used for investigating the correlations. A one-way ANOVA with a Tukey's post hoc test (**A** and **B**) or Spearman's rank correlation coefficient (**C**) was used for statistical analysis. $P<0.05$ was considered significant.

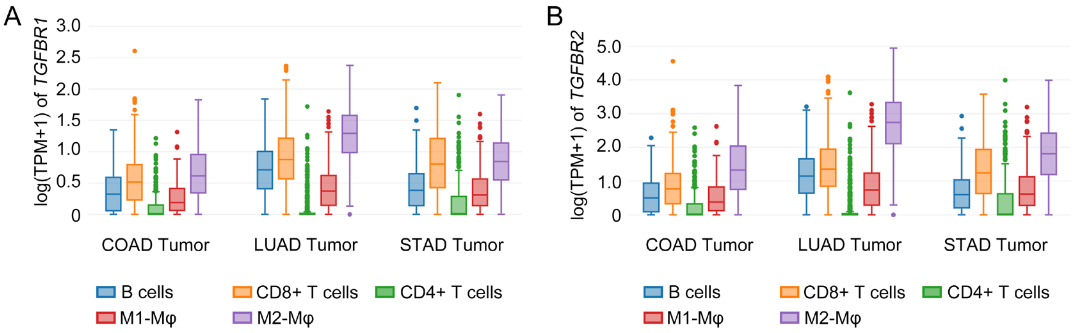

Figure S3. *TGFBR1* and *TGFBR2* are preferentially expressed by infiltrating M2-Mφ in common human tumors. (**A** and **B**) *TGFBR1* and *TGFBR2* gene expression in the infiltrating immune cells of common human tumors (COAD, colon adenocarcinoma; LUAD, lung adenocarcinoma; STAD, stomach adenocarcinoma; based on GEPIA platform, http://gepia2021.cancer-pku.cn/index.html). A one-way ANOVA with a Tukey's post hoc test was used for statistical analysis.

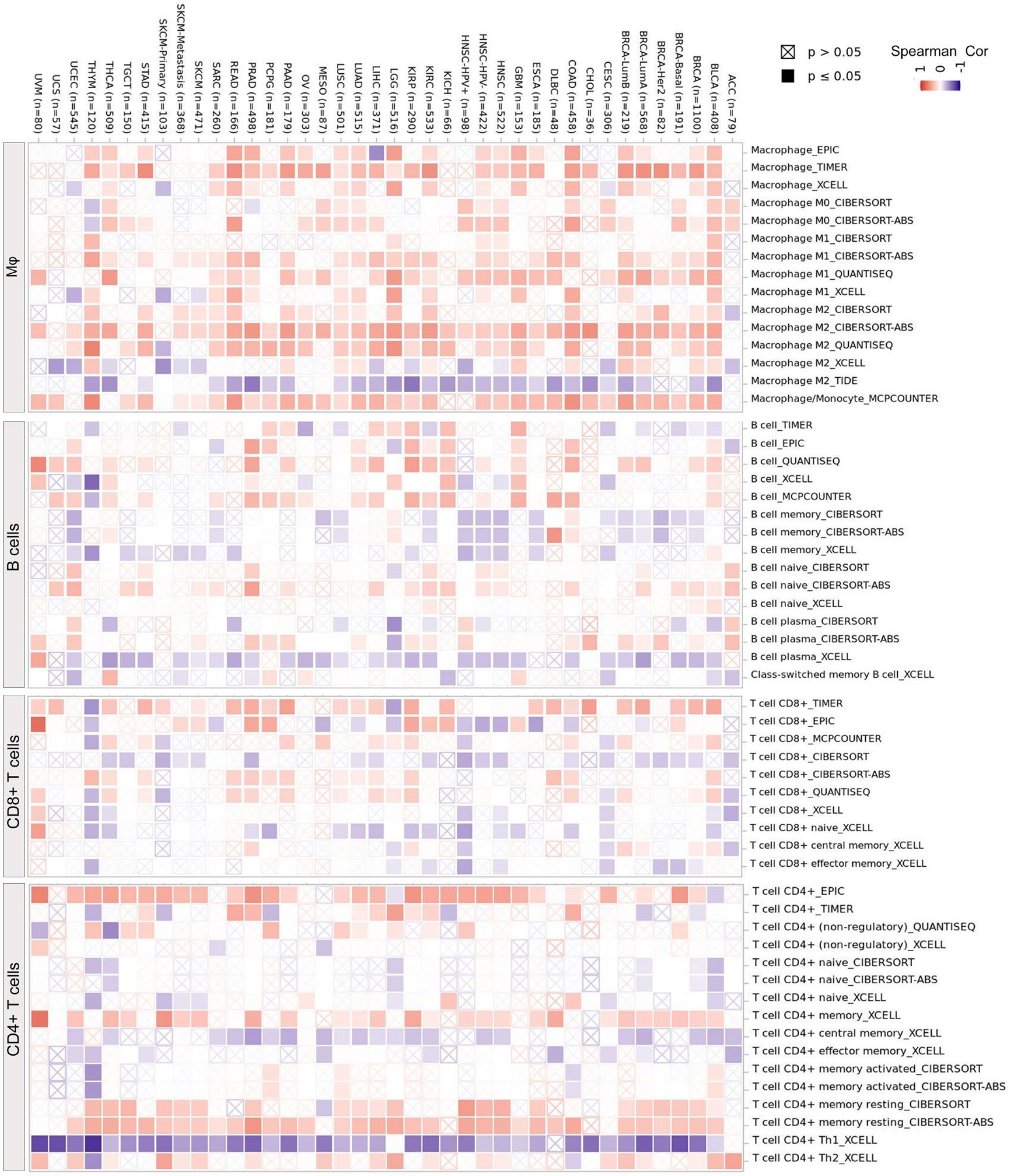

Figure S4. Correlations between *TGFBR1* gene expression and infiltrating immune cell types. Correlations analysis was performed in various human tumors by the TIMER web server using different algorithms (TIMER2.0; https://cistrome.shinyapps.io/timer/). Abbreviations for various human tumors are given according to the database. Correlation (Cor) was determined by Spearman's rank coefficient. *P*<0.05 was considered significant.

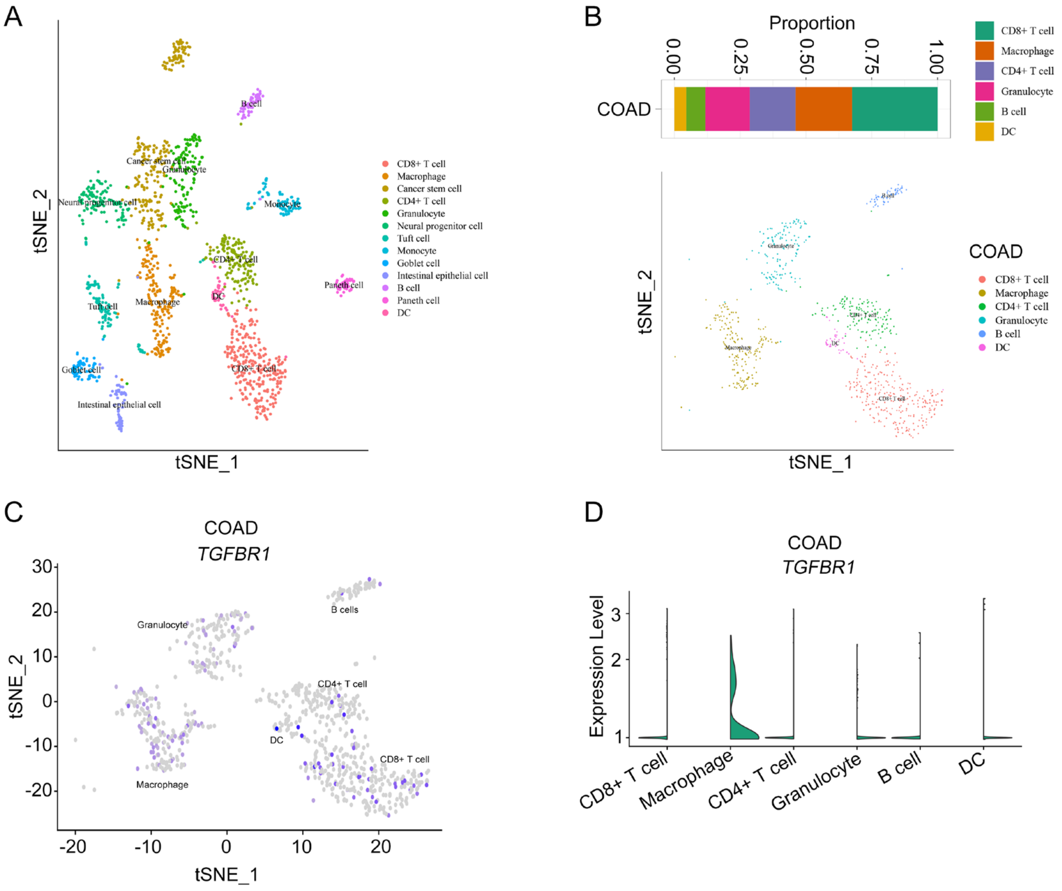

Figure S5. *TGFBR1* is preferentially expressed by infiltrating Mφ in human colon adenocarcinoma. A publicly available scRNA-seq dataset of human colon cancer samples (GSE161277) was analyzed. (**A**) tSNE plot showing major cell types of the tumor sample. (**B**) The proportion of infiltrating immune cells (upper) and their clusters in the tSNE plot (lower). (**C** and **D**) *TGFBR1* expression for infiltrating immune cells was visualized using tSNE (**C**) and violin plots (**D**). The coloring (blue) depicts *TGFBR1* levels in tSNE islands.

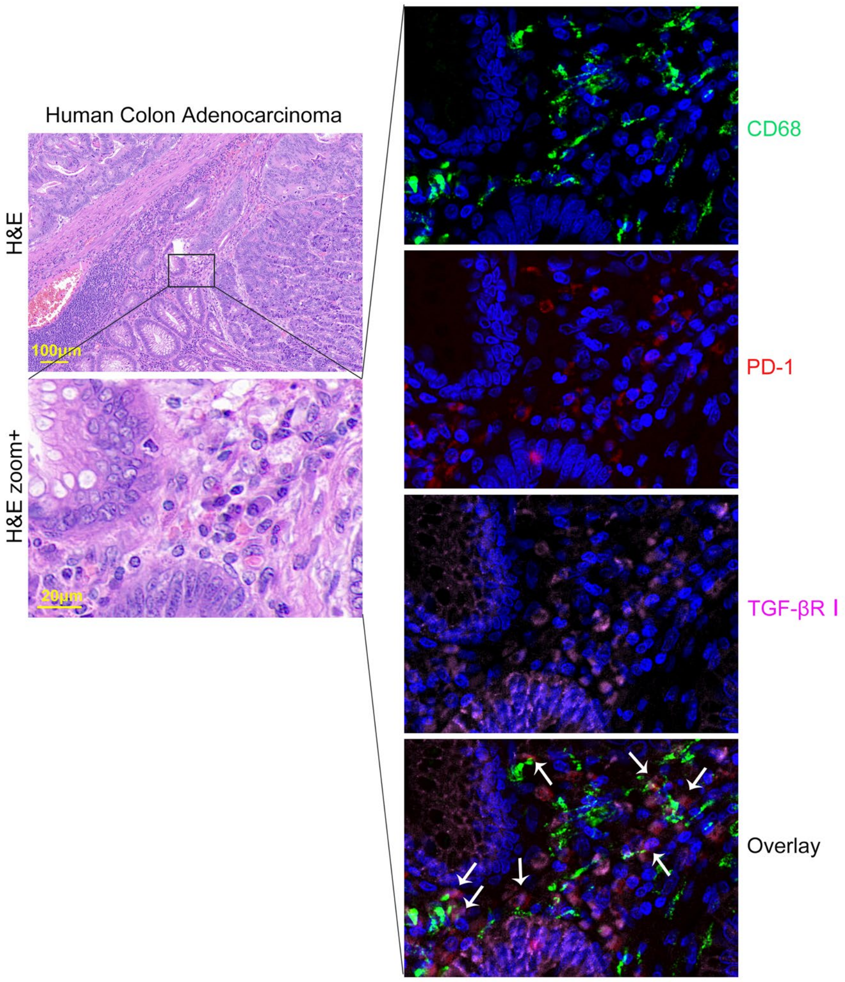

Figure S6. Representative immunofluorescence co-staining of CD68, PD-1, TGF-βRI, and DAPI in one human colon cancer specimen. Arrowheads in the merged image show the co-expression of PD-1 and TGF-βRI in $CD68^{+}$ Mφ. Scale bars, 100 or 20 μm.

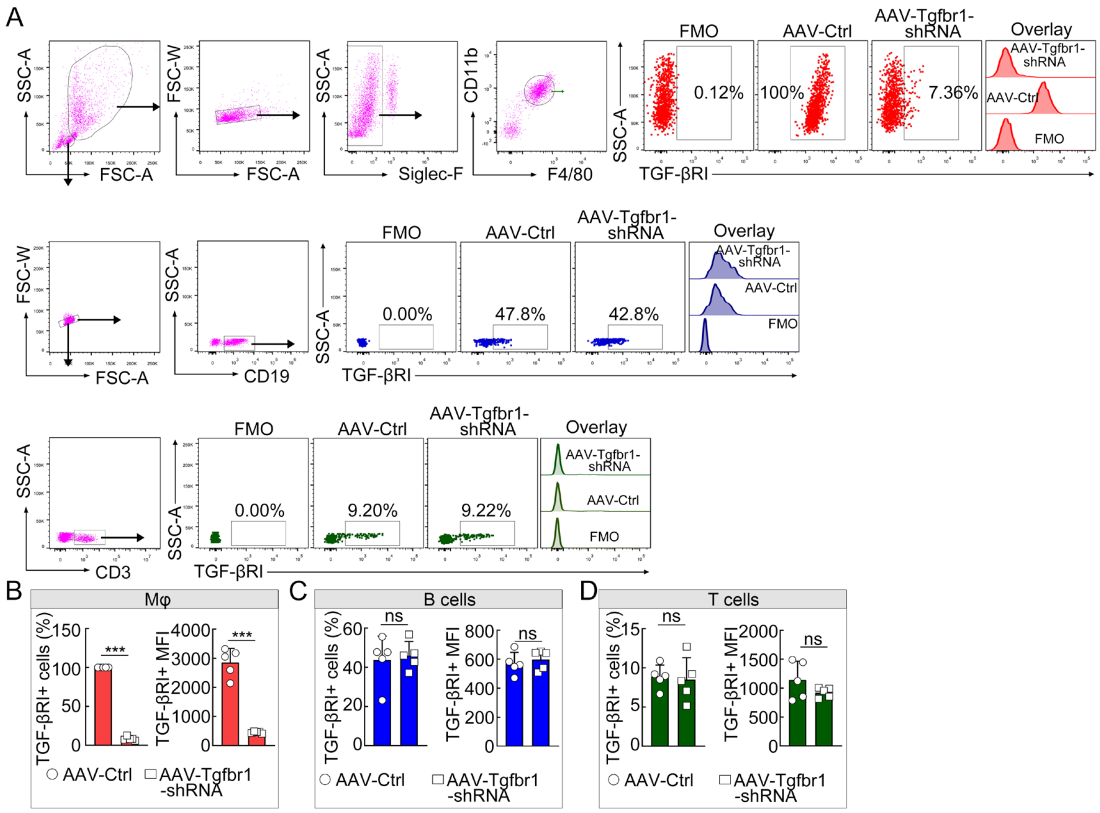

Figure S7. The efficiency of Mφ-specific TGF-βRI-knockdown. Mice were injected intraperitoneally with AAV-F4/80-mirR30-shTgfbr1 or the control vector. Resident peritoneal cells were collected. TGF-βRI expression on peritoneal CD11b$^+$F4/80$^+$ Mφ, CD19$^+$ B cells, and CD3$^+$ T cells were analyzed by FCM. The respective representative dot plots and histograms (**A**), and quantification graphs of percentages and MFI (**B**-**D**) of TGF-βRI expression are shown. An unpaired *t* test (**B**-**D**) was used for statistical analysis. All graph data are expressed as the mean ± SD of 5 mice. ns indicating not significant, ***P<0.001.

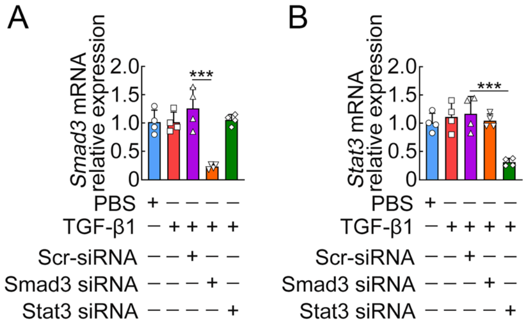

Figure S8. The silencing efficiency of siRNA knockdown. RAW 264.7 Mφ were transfected with scrambled (Scr)-siRNA, Smad3-specific siRNA, or Stat3-specific siRNA. Cells were collected at 24 hours after siRNA transfection. Relative *Smad3* mRNA (**A**) and *Stat3* (**B**) mRNA expression levels were determined using RT-PCR. A one-way ANOVA with a Tukey's post hoc test (**A** and **B**) was used for statistical analysis. All graph data are expressed as the mean ± SD of 4 biological replicates per group. ****P*<0.001.
