# Supplementary material for: TGF-β1 induces PD-1 expression in macrophages through SMAD3/STAT3 cooperative signaling in chronic inflammation
Source: JCI Insight. 2024 Mar 5;9(7):e165544. doi: 10.1172/jci.insight.165544 (PMC11128204; doi:10.1172/jci.insight.165544)

Lanes used in Figure

Full unedited gel for Figure 2C

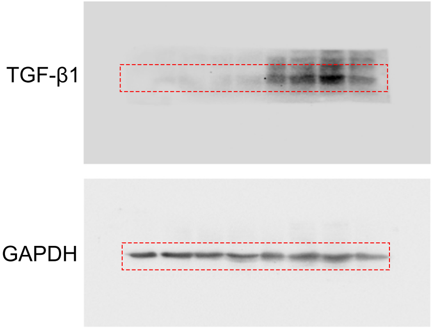

Full unedited gel for Figure 2D

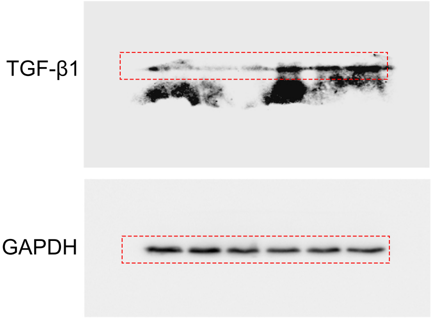

Full unedited gel for Figure 5J

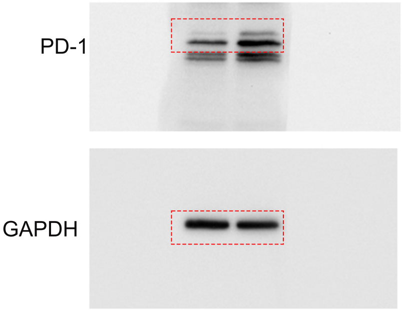

Full unedited gel for Figure 6A

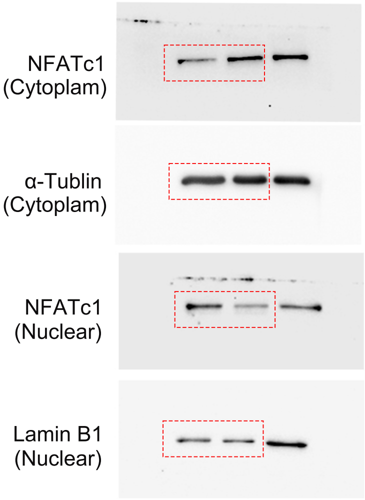

Full unedited gel for Figure 6C

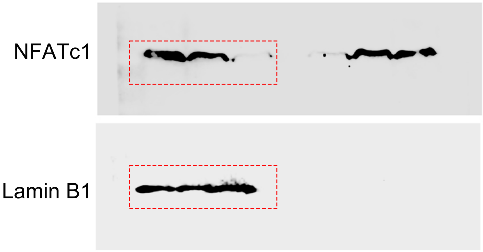

Full unedited gel for Figure 6F

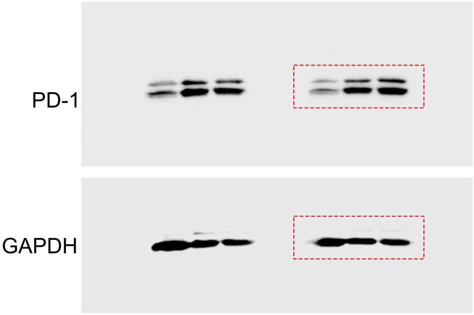

Full unedited gel for Figure 7G

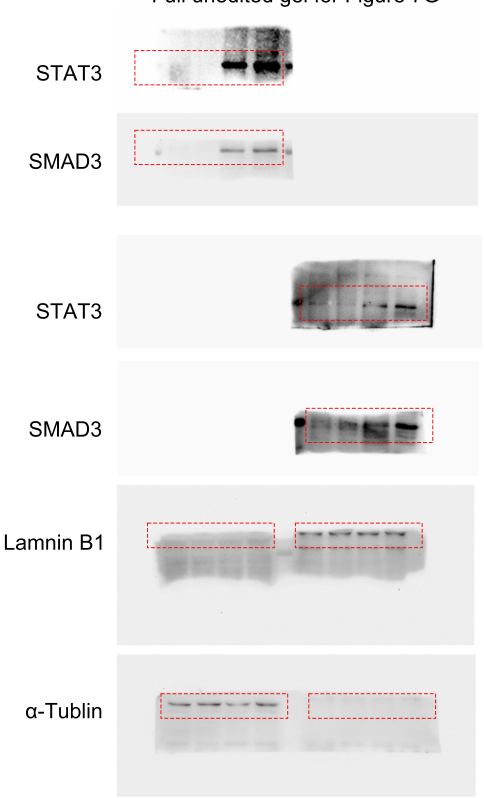

Full unedited gel for Figure 8A

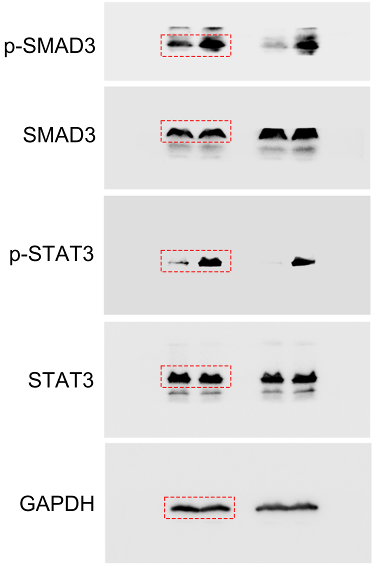

Full unedited gel for Figure 8C

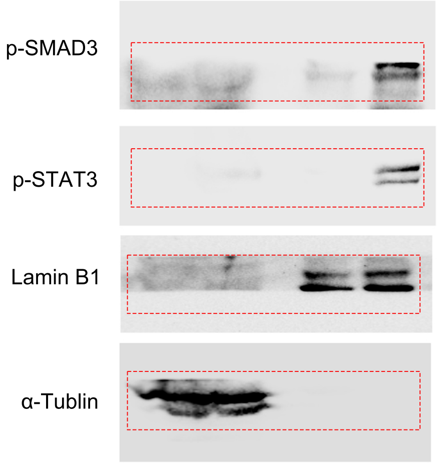

Full unedited gel for Figure 8E

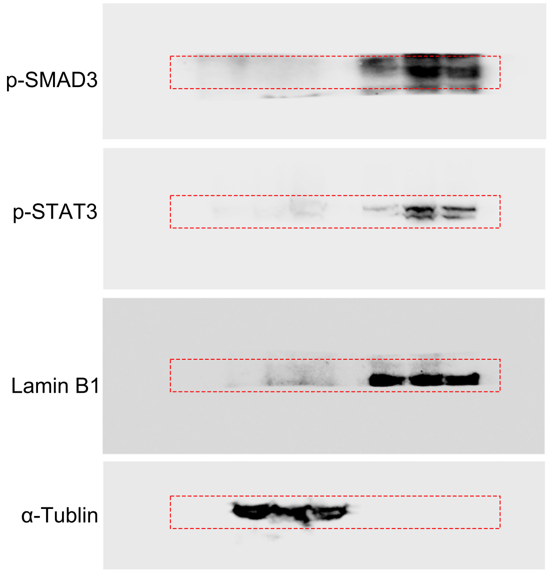

Full uneidted gel for Figure 8M

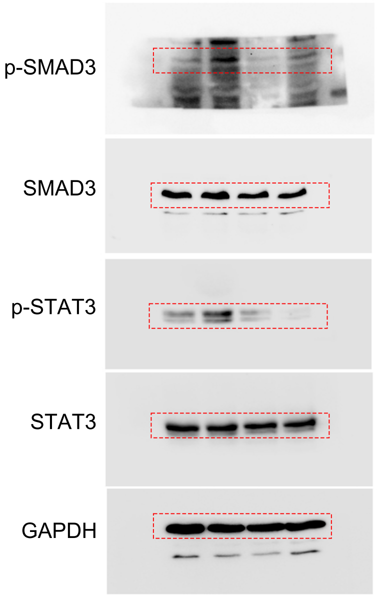

Supplement: Unedited blot and gel images [file jciinsight-9-165544-s171.pdf]
